# LRRK2 kinase activity regulates Parkinson’s disease-relevant lipids at the lysosome

**DOI:** 10.1186/s13024-025-00880-7

**Published:** 2025-08-06

**Authors:** Michael T. Maloney, Xiang Wang, Rajarshi Ghosh, Shan V. Andrews, Romeo Maciuca, Shababa T. Masoud, Maayan Agam, Richard M. Caprioli, Giuseppe Astarita, Vitaliy V. Bondar, John Chen, Chi-Lu Chiu, Sonnet S. Davis, Audrey Cheuk-Nga Ho, Hoang N. Nguyen, Nicholas E. Propson, Michelle L. Reyzer, Oliver B. Davis, Matthew C. Deen, Sha Zhu, Gilbert Di Paolo, David J. Vocadlo, Anthony A Estrada, Javier de Vicente, Joseph W. Lewcock, Annie Arguello, Jung H. Suh, Sarah Huntwork-Rodriguez, Anastasia G. Henry

**Affiliations:** 1 Denali Therapeutics Inc, 161 Oyster Point Blvd, South San Francisco, CA 94080 USA; 2 Mass Spectrometry Research Center, Vanderbilt University, 9160 MRB III, 465 21 Avenue South, Nashville, TN 37240 USA; 3 Department of Chemistry, Simon Fraser University, Burnaby, BC V5A 1S6 Canada; 4 Tenvie Therapeutics, South San Francisco, CA 94080 USA

**Keywords:** LRRK2, Lysosome, Parkinson’s disease, BMP and glycosphingolipids

## Abstract

**Background:**

Pathogenic variants in *LRRK2* lead to increased kinase activity, and LRRK2 kinase inhibition is being explored in clinical studies as a therapeutic approach for Parkinson’s Disease (PD). LRRK2 inhibitors reduce urine levels of bis(monoacylglycerol)phosphate (BMP), a key endolysosomal lipid involved in glycosphingolipid (GSL) catabolism, in preclinical models and clinical subjects. However, how LRRK2 regulates BMP and its significance with respect to lysosomal dysfunction in PD are poorly defined.

**Methods:**

Using a combination of genetic and pharmacological approaches to modulate LRRK2 kinase activity, we explored the mechanisms by which LRRK2 can regulate the levels of BMP and PD-relevant GSLs across cellular models, including iPSC-derived microglia, and in tissues and biofluids from mice using mass spectrometry. The impact of LRRK2 activity on various aspects of lysosomal function, including endolysosomal GCase activity, was assessed using live-cell imaging and lysosomal immunoprecipitation. We employed imaging mass-spectrometry and FACS-based methods to specifically examine how LRRK2 modulates BMP and GSL levels across different cell types and regions of the brain. To confirm the relevance of our findings to disease, we measured lysosomal biomarkers in urine and cerebrospinal fluid (CSF) from human subjects carrying variants in LRRK2 associated with PD risk and from subjects dosed with a LRRK2 kinase inhibitor.

**Results:**

Our data demonstrate that LRRK2 can employ distinct mechanisms to control intracellular BMP levels and modulate lysosomal homeostasis depending on the tissue examined. We show that LRRK2 deletion or inhibition lowers urine BMP levels by reducing the secretion of BMP-containing vesicles from kidney into urine. In other cell types such as microglia, LRRK2-mediated inhibition of β-glucocerebrosidase (GCase), a PD-linked enzyme involved in GSL catabolism, leads to lysosomal GSL accumulation and increases BMP levels as a compensatory response to restore lysosomal homeostasis. LRRK2 inhibition normalizes lysosomal function and reduces GSL levels in preclinical models and CSF from LRRK2-PD patients.

**Conclusions:**

Our study highlights the therapeutic potential of LRRK2 kinase inhibition to improve PD-associated lysosomal dysfunction and supports the utility of GSLs as CSF-based biomarkers of LRRK2 activity.

**Trial registration:**

This work includes results from the following phase 1b study in PD patients: ClinicalTrials.gov ID: NCT03710707; https://clinicaltrials.gov/study/NCT03710707?intr=dnl201&rank=2. The date of registration was 10/18/2018.

**Supplementary Information:**

The online version contains supplementary material available at 10.1186/s13024-025-00880-7.

## Background

Lysosomal dysfunction has emerged as a principal contributor to the susceptibility and pathogenesis of several neurodegenerative diseases including PD. Whole exome sequencing and genome-wide association studies for PD have converged on genes that function in the autophagic and endolysosomal pathways such as *LRRK2*, *GBA1*, and *TMEM175* [1–5]. Moreover, an increased burden of variants in genes that cause rare monogenic lysosomal storage disorders (LSDs) has been observed in PD patients relative to controls, suggesting common mechanisms of lysosomal dysfunction drive both severe LSDs and PD [6–8]. Defects in lysosomal homeostasis have also been seen in PD patient postmortem brain samples or biofluids, including reduction of the lysosomal compartment and GSL storage [9–13]. These data suggest that approaches aimed at correcting lysosomal dysfunction may provide new therapeutic avenues for the treatment of PD, for which there remains no disease-modifying therapy.

LRRK2 kinase inhibition is currently being explored in late-stage clinical studies as one such approach to improve lysosomal function in PD [14, 15]. Variants in *LRRK2* are associated with increased risk for Parkinson’s and Crohn’s disease (CD), including a putative protective haplotype (N551K-R1398H) linked to reduced risk for both PD and CD [16–18]. *LRRK2* variants are proposed to modify PD risk by regulating the enzyme’s kinase activity, as most pathogenic variants lead to increased kinase activity, and emerging evidence suggests that the protective variant is associated with reduced activity [19–21]. LRRK2 phosphorylates a subset of Rab GTPases, known master regulators of the secretory and endocytic pathways, and PD risk-linked *LRRK2* variants increase phosphorylation of these Rab substrates, including Rab10 and Rab12 [22, 23]. This phosphorylation is thought to impair Rab function by perturbing interactions with downstream effectors, resulting in defects in various aspects of intracellular trafficking and dysfunction in the autophagic and endolysosomal system. LRRK2 has been shown to act as both a sensor and trigger of lysosomal dysfunction as the kinase localizes to endolysosomal membranes upon damage in a Rab12-dependent fashion and can modulate autophagic flux and lysosomal proteolysis [24–28]. The detailed mechanisms by which LRRK2 regulates lysosomal function and the relevance of this dysfunction to PD, however, are still not clear.

One potential mechanism by which LRRK2 can modulate lysosomal homeostasis may be through its established connection with BMP, an anionic phospholipid concentrated on intraluminal vesicles (ILVs) in late endosomes and lysosomes [29, 30]. LRRK2 kinase inhibition has been previously shown to reduce urine BMP levels in preclinical models and in human subjects [14, 31], and carriers of the *LRRK2* G2019S variant associated with increased kinase activity have higher levels of BMP in their urine compared to non-carriers [32]. These results demonstrate that LRRK2 activity regulates the extracellular concentration of BMP in urine; however, it is difficult to interpret what such changes mean with respect to LRRK2’s impact on BMP levels in tissues and its consequences on lysosomal function more broadly. BMP promotes the hydrolysis of GSLs by serving as an anionic lipid anchor and stimulator of lysosomal lipid hydrolase activities, including the PD-linked enzyme GCase encoded by *GBA1* [33, 34], and increasing data suggest that defective GSL metabolism may play a key role in PD pathogenesis [35]. Collectively, this suggests that LRRK2 kinase activity may impact lysosomal function through its action on BMP and contribute to impaired GSL homeostasis associated with PD. It remains to be determined whether LRRK2 regulates BMP or GSL levels in the brain, and the potential value of these lipids as CSF-based biomarkers of LRRK2-dependent effects on lysosomal function has yet to be assessed. Accordingly, a deeper understanding of the mechanisms by which LRRK2 regulates BMP and its impact on lysosomal function is needed to evaluate the therapeutic potential of LRRK2 kinase inhibition in PD and determine the utility of BMP and GSLs as lysosomal biomarkers of LRRK2 activity in the clinic.

Here, we define the mechanisms underlying LRRK2-dependent regulation of BMP levels and show that LRRK2-mediated changes in BMP correlate with GSL dysregulation and lysosomal dysfunction in preclinical models and PD patients. First, our data suggest that LRRK2 activity regulates the levels of BMP in urine by promoting the secretion of BMP-containing vesicles from kidney, providing mechanistic context to enable the use of urinary BMP as a biomarker of LRRK2’s effects on lysosomal function in the periphery. In contrast to effects observed in kidney, we demonstrate that LRRK2 hyperactivity increases levels of BMP in lysosomes in other cell types, including in the CNS, and show that this occurs as a secondary consequence of LRRK2-mediated effects on GCase activity. Hyperactive LRRK2 impairs endolysosomal GCase activity and leads to GSL accumulation, while LRRK2 kinase inhibition reduces BMP and GCase substrate levels in lysosomes and ultimately restores lysosomal function. Finally, our results indicate that GSLs, including glucosylceramide (GlcCer) and galactosylceramide (GalCer), may serve as potential treatment-responsive biomarkers of LRRK2 inhibition in the CNS based on our analysis of CSF from LRRK2-PD patients dosed with a LRRK2 inhibitor in a phase 1b study. Together, these results support the exploration of BMP and GSLs as biomarkers of LRRK2-dependent regulation of lysosomal function in ongoing clinical studies and highlight the therapeutic potential of LRRK2 inhibition to correct lysosomal dysfunction observed in PD.

**Fig. 1:**
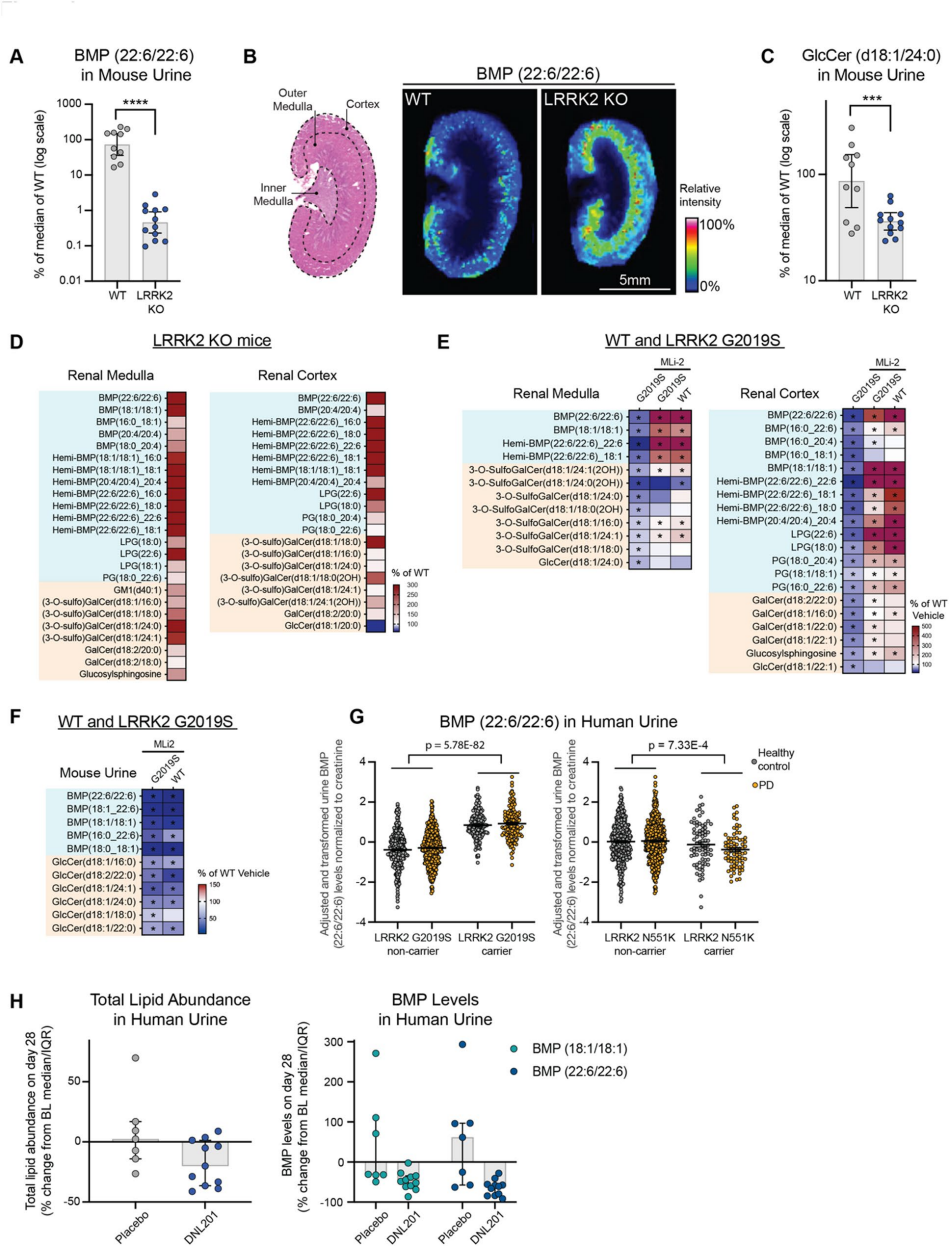
LRRK2 activity regulates peripheral BMP levels in preclinical models and in human subjects. **A**) Urine BMP(22:6/22:6) levels were measured from LRRK2 KO mice (*n* = 12) and wildtype (WT) littermates (*n* = 10). The relative abundance of urine BMP (22:6/22:6) levels were normalized to creatine, measured by LC-MS/MS, and then presented as a percent of median values measured in the WT group. Data are shown as geometric mean ratio percent and 95% confidence intervals, with statistical significance assessed based on Benjamini-Hochberg-adjusted p-values; ****p ≤ 0.0001. **B**) Matrix-assisted laser desorption/ionization mass spectrometry images were acquired from longitudinal kidney sections of WT and LRRK2 KO mice. Left panel: Representative hematoxylin and eosin photomicrograph of the kidney showing demarcated regions (cortex, outer medulla and inner medulla). Right panel: Representative mass spectrometry image showing the distribution of the signal at a mass/charge (m/z) ratio of 865.502, corresponding to BMP(22:6/22:6). Images depict the relative intensity of the signal from 0 to 100% with intensity normalized using total ion current. **C**) GlcCer(d18:1/24:0) levels in urine from LRRK2 KO mice (*n* = 12) and WT littermates (*n* = 10) were measured by LC-MS/MS and then presented as a percent of median values measured in the WT group. Data are shown as geometric means with 95% CI, with p-values based on an ANCOVA model with statistical significance assessed using on Benjamini-Hochberg (BH)-adjusted p-values; ***p ≤ 0.001. **D**) Heatmap showing the relative abundance of a panel of lipid species measured in the renal cortex and medulla from LRRK2 KO mice compared to WT littermates measured using LC-MS/MS; *n* = 11–12 animals for each group. The heatmap was generated as percent of change by normalizing the average of LRRK2 KO mice to the average of the WT group. The analytes included had nominal p-values (*p ≤ 0.10) for genotype differences and were grouped based on lipid class. White in the color scale depicts the WT-vehicle amounts, as 100%; red shows an accumulation (capped at 300%), and blue shows a reduction. **E** and **F**) LRRK2 G2019S knock-in (KI) mice and WT littermates were administered either vehicle or the tool LRRK2 kinase inhibitor MLi-2 (100 mg/kg) in chow, and a panel of lipid species were measured in renal cortex and renal medulla and in urine after 35 days of dosing using LC-MS/MS; *n* = 12 for WT-vehicle group, *n* = 12 for WT-MLi-2 group, *n* = 13 for LRRK2 G2019S-vehicle group, and *n* = 14 for LRRK2 G2019S-MLi-2 group. Heatmaps were generated as percent of change by normalizing the average of different groups to the average of the WT vehicle group. The analytes included had nominal p-values (* *p* ≤ 0.10) for genotype differences and were grouped based on lipid class. * *p* ≤ 0.10 annotated in the MLi-2 treatment groups are based on the MLi-2 vs. vehicle comparisons within the same genotype. White in the color scale depicts the WT-vehicle amounts, as 100%; red shows an accumulation (capped at 500% in panel E, capped at 150% in panel F), and blue shows a reduction. **G**) Association of urine BMP(22:6/22:6) levels and carrier status at the PD-risk LRRK2 G2019S variant and the PD-protective LRRK2 N551K variant in PPMI data. Urine BMP levels were normalized to creatinine levels, log transformed, and fit in a linear model against sex, age, disease status, and the first five principal components derived from whole genome sequencing data. Inverse normal transformed residuals from this linear model are plotted on the y-axis and used in association testing with LRRK2 variant status. Data are shown as mean ± SEM. **H**) Reduction of total lipid and BMP levels in urine exosomes collected from human subjects treated with DNL201 (*n* = 11) compared with the placebo group (*n* = 7). The total lipid abundance was calculated by summing up all the abundance of all the lipids analyzed. The percentage of changes from baseline (BL) was analyzed by calculating the lipid abundance change from the day 28-post-dose to pre-dose-baseline, and then normalizing the change to lipid abundance at pre-dose baseline. Lipid levels are expressed as percent change from pre- to post- dose, and data are shown as median with interquartile range (IQR)

**Fig. 2:**
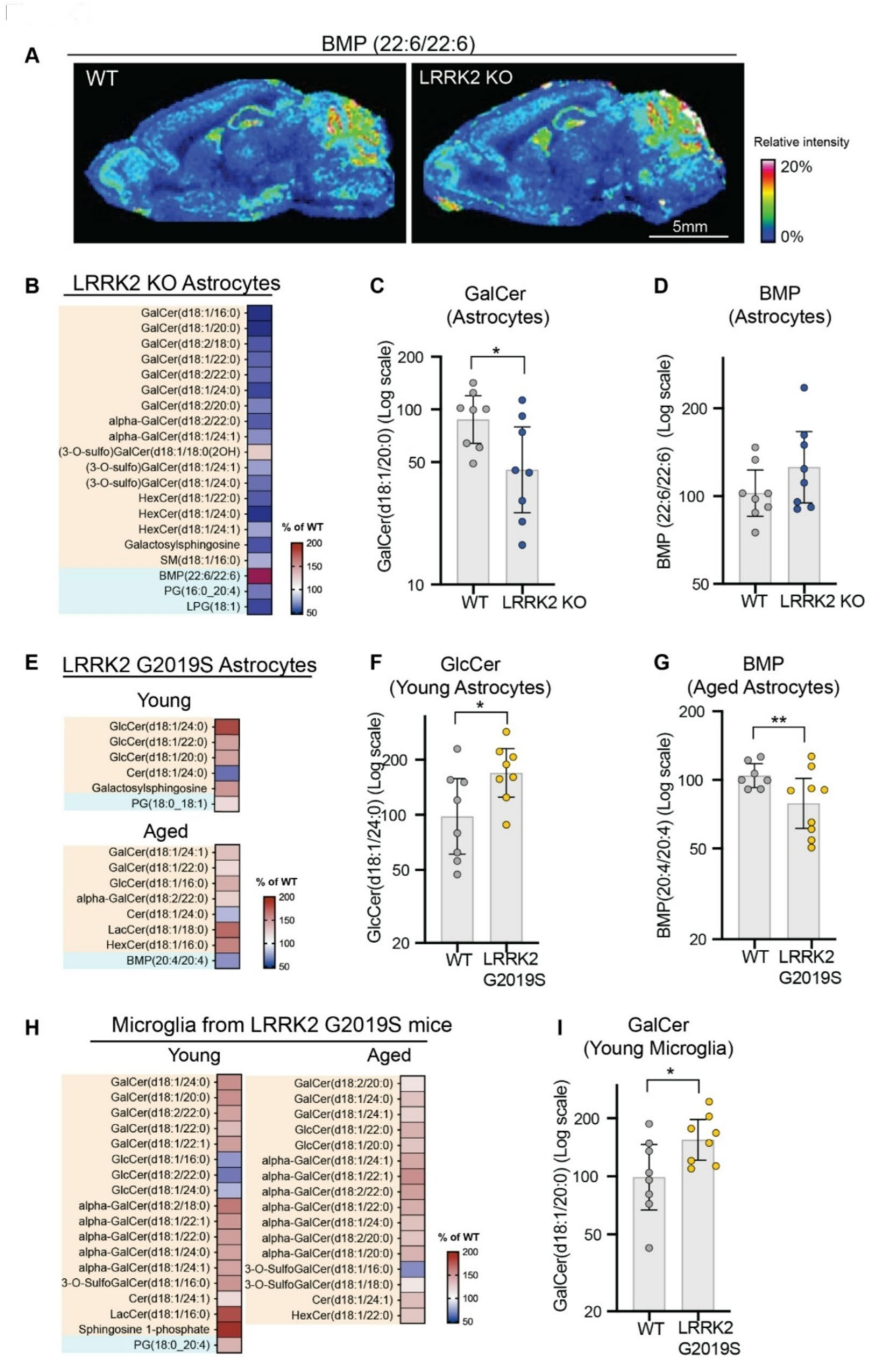
LRRK2 activity regulates glycosphingolipids in mouse brain and mildly impacts BMP. **A**) Matrix-assisted laser desorption/ionization mass spectrometry images were acquired from sagittal brain sections of WT and LRRK2 KO mice. Representative mass spectrometry image showing the distribution of the signal at a mass/charge (m/z) ratio of 865.502, corresponding to BMP(22:6/22:6). Images depict the relative intensity of the signal from 0 to 20% with intensity normalized using total ion current. **B**) A heatmap representing the percent change in the levels of BMP-related lipids and GSLs measured in astrocytes isolated from LRRK2 KO mice compared to WT mice; *n* = 8 for LRRK2 KO mice and WT littermates. **C** and **D**) The levels of GalCer(d18:1/20:0) and BMP(22:6/22:6) were quantified from astrocytes isolated from LRRK2 KO mice and WT littermates. E-H) Astrocytes and microglia were isolated from young (5–6 month-old) and aged (18 month-old) LRRK2 G2019S KI mice and WT littermates, and their lipid profiles were assessed using LC-MS/MS; *n* = 8 for young LRRK2 G2019S KI and WT littermates and *n* = 7 for aged WT littermates and *n* = 9 for aged LRRK2 G2019S KI mice. **E**) Heatmaps representing the percent change in the levels of BMP-related lipids and GSLs measured in astrocytes isolated from young and aged LRRK2 G2019S KI mice compared to WT littermates. **F** and **G**) The levels of GlcCer(d18:1/24:0) and BMP(20:4/20:4) were quantified from astrocytes isolated from LRRK2 G2019S mice and WT littermates. **H**) Heatmaps representing the percent change in the levels of BMP-related lipids and GSLs measured in microglia isolated from young and aged LRRK2 G2019S KI mice compared to WT littermates. **I**) The levels of GalCer(d18:1/20:0) were quantified from microglia isolated from young LRRK2 G2019S mice and WT littermates. The heatmaps (**B**, **E** and **H**) were generated as percent change compared to levels measured in WT littermate controls. Shown are analytes with nominal p-values (*p ≤ 0.10) for genotype difference that were grouped based on lipid class. The BMP-related lipids were shaded in cyan, and GSL species were shaded in orange. In the color scale, white depicts the WT amounts, set to 100%, red shows an accumulation (capped at 200%) and blue shows a reduction. For plots (**C**, **D**, **F**, **G** and **I**), the relative abundance of the analytes was normalized to the median values of WT group. Data are plotted in log scale and shown as geometric mean ratio percent and 95% CIs with statistical significance assessed at nominal levels. *p ≤ 0.05, **p ≤ 0.01

**Fig. 3:**
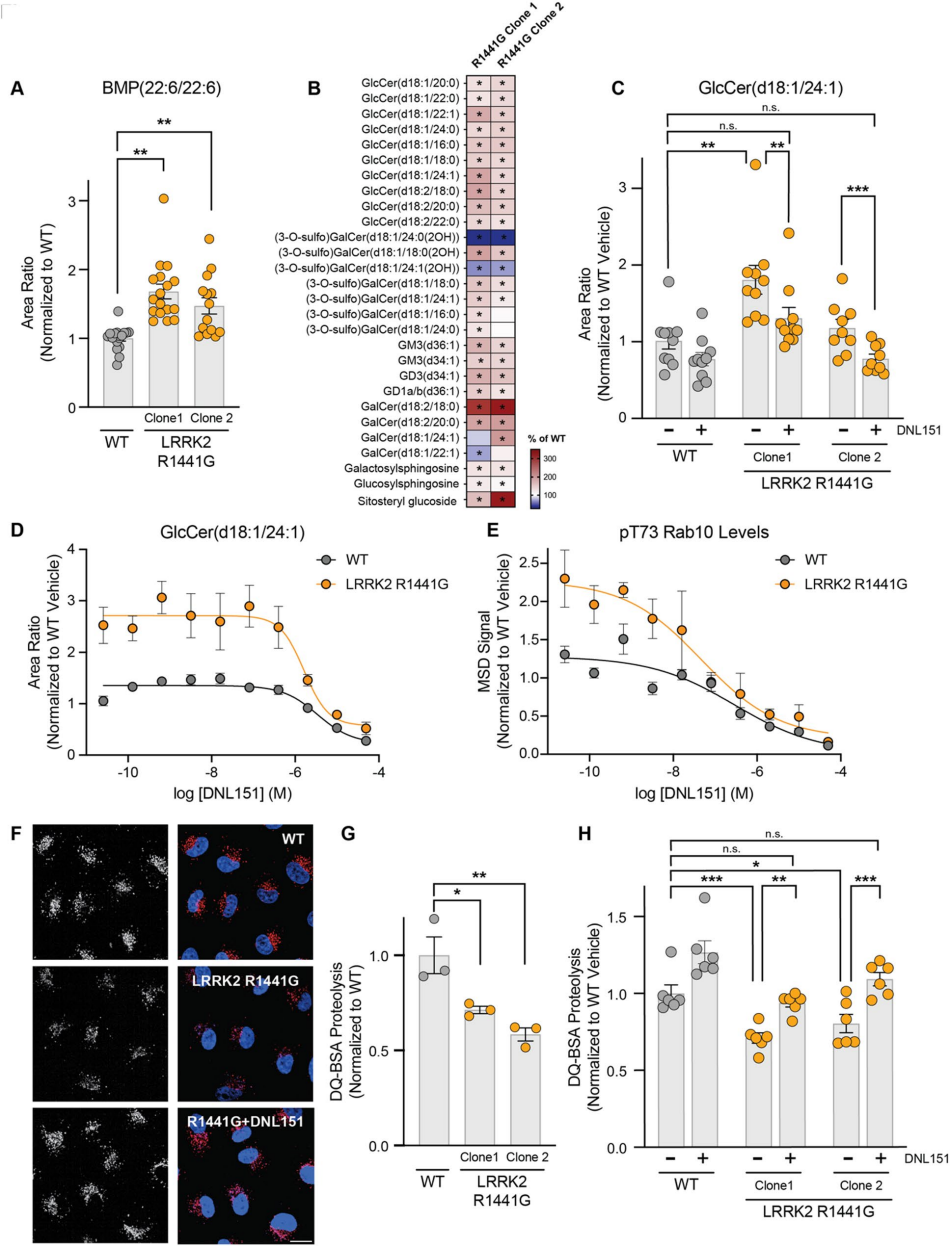
LRRK2 activity regulates glucosylceramide and BMP levels to maintain proper lysosomal function. **A**) BMP(22:6/22:6) levels were measured in WT parental A549 cells and two clones of LRRK2 R1441G KI A549 cells; data are shown as mean ± SEM; *n* = 17 independent experiments, and statistical significance was determined using one-way ANOVA following log transformation. **B**) Heatmap showing elevated glycosphingolipids including multiple GlcCer species in whole cell extracts from two clones of LRRK2 R1441G KI A549 cells as compared to parental WT cells. The heatmaps were generated as percent of change by normalizing the average of different groups to the average of the WT group; *n* = 14 independent experiments. The analytes included had nominal p-values (*p ≤ 0.10) for genotype difference in either clone and were grouped based on lipid class. White in the color scale depicts the WT-vehicle as 100%, red shows an accumulation (capped at 350%), and blue shows a reduction. **C**) WT and two clones of LRRK2 R1441G KI A549 cells were treated with vehicle or DNL151 (2µM) for 72 h, and the levels of GlcCer(d18:1/24:1) were measured using LC-MS/MS. Data are shown as mean ± SEM; *n* = 10 independent experiments, and statistical significance was determined using one-way ANOVA following log transformation. **D**) WT and one clonal line of LRRK2 R1441G KI A549 cells were treated with increasing concentrations of DNL151, and the levels of GlcCer(d18:1/24:1) were measured using LC-MS/MS. Data are shown as mean ± SEM; *n* = 4 independent experiments, and statistical significance was determined using non-linear fit following log transformation. **E**) Dose-response curves show the percent inhibition of LRRK2 kinase activity as measured by levels of phosphorylated T73 Rab10 in WT and one clonal line of LRRK2 R1441G A549 cells. Data are shown as mean ± SEM; *n* = 4 independent experiments, and statistical significance was determined using non-linear fit following log transformation. **F**) Representative images of DQ-BSA signals (left panels: black and white, right panels: red) in WT A549 cells (top panel), LRRK2 R1441G KI cells (middle panel) and LRRK2 R1441G KI cells treated with DNL151(bottom panel). Nuclei stained with NucBlue (blue); scale bar = 10 μm. **G**) The sum of spot intensities of DQ-BSA signal was quantified per cell in WT and two clones of LRRK2 R1441G KI A549 cells. The DQ-BSA signals were normalized to the median within each experiment and to the WT control. Data are shown as mean ± SEM; *n* = 3 independent experiments, and statistical significance was determined using one-way ANOVA following log transformation, *p ≤ 0.05, **p ≤ 0.01. **H**) WT and two clones of LRRK2 R1441G KI A549 cells were treated with vehicle or DNL151 (2µM) for 72 h, and lysosomal proteolysis was measured using the DQ-BSA-based assay. The sum spot intensities of DQ-BSA signal were quantified per cell. The DQ-BSA signals were normalized to the median within each experiment and to the WT vehicle control. Data are shown as mean ± SEM; *n* = 6 independent experiments, and statistical significance was determined using one-way ANOVA following log transformation. *p ≤ 0.05, **p ≤ 0.01, ***p ≤ 0.001

**Fig. 4:**
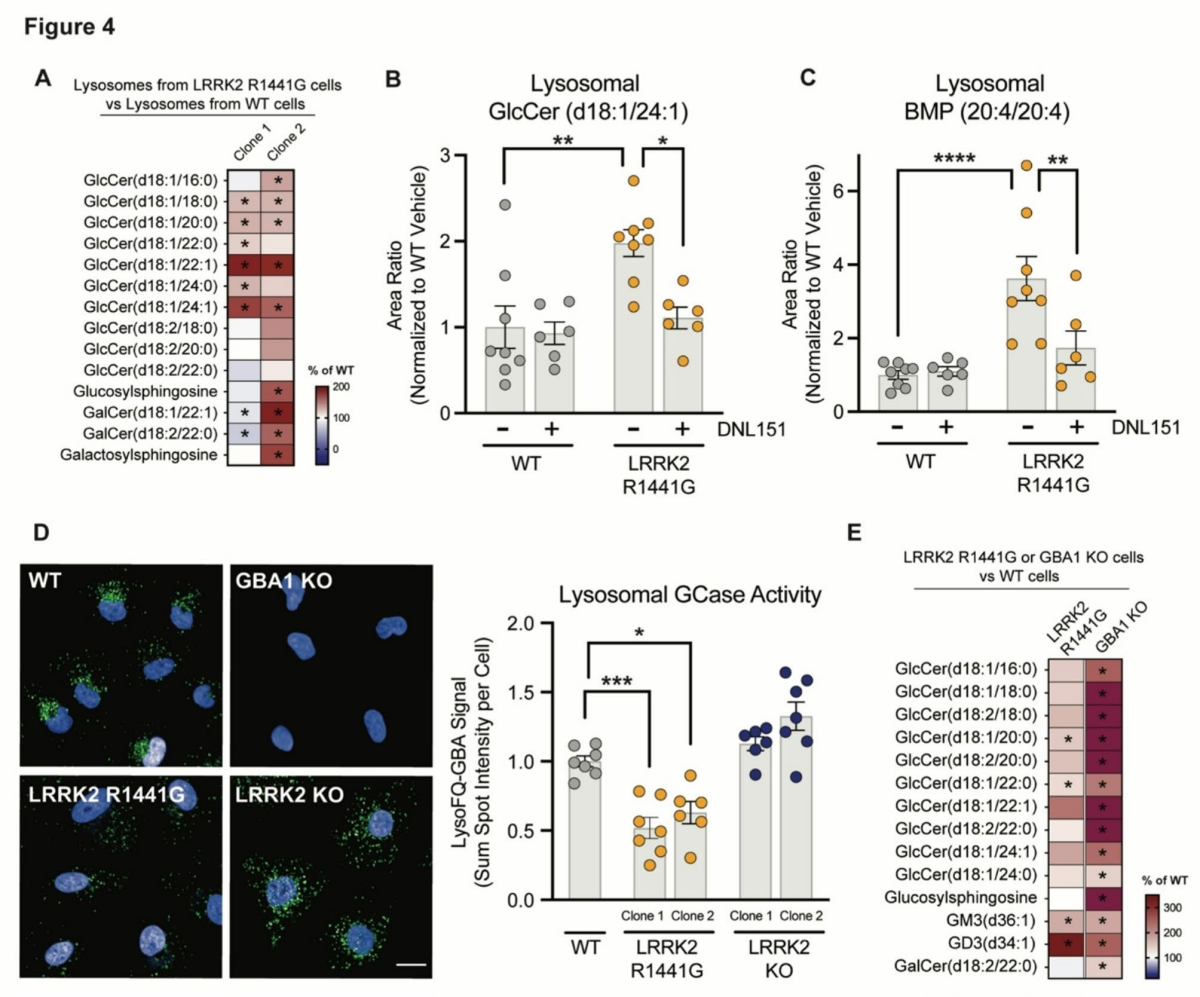
LRRK2 activity regulates endolysosomal GCase activity and GlcCer and BMP. levels in lysosomes. **A**) The levels of GSL species were measured in lysosomes isolated from WT and two clones of LRRK2 R1441G KI A549 cells. The percent change in signal from LRRK2 R1441G cells compared to WT cells is shown in the heatmap. The analytes included had nominal p-values (* *p* ≤ 0.10) for genotype difference and were grouped based on lipid class; *n* = 12 independent experiments. White in the color scale depicts the WT-vehicle as 100%, red shows an accumulation (capped at 200%), and blue shows a reduction. **B** and **C**) WT and one clonal line of LRRK2 R1441G KI A549 cells were treated with vehicle or DNL151 (2µM) for 72 h, lysosomes were rapidly immunoprecipitated, and the levels of GlcCer(d18:1/24:1) or BMP(20:4/20:4) were measured using LC-MS/MS. Data are shown as mean ± SEM; *n* = 8 independent experiments, and statistical significance was determined using one-way ANOVA following log transformation. **D**) Left: Representative images of GCase activity in WT, GBA1 KO, LRRK2 R1441G KI and LRRK2 KO A549 cells as shown by LysoFQ-GBA probe fluorescence (green). Nuclei stained with NucBlue (blue); scale bar = 10 μm. Right: Quantification of GCase activity as measured by the LysoFQ-GBA probe fluorescence signal. The sum of spot intensities of the LysoFQ-GBA signal was quantified per cell from WT, two clones of LRRK2 R1441G KI and two clones of LRRK2 KO A549 cells and was normalized to the median within each experiment and to the average of the WT controls across experiments. Data are shown as mean ± SEM; *n* = 7 independent experiments, and statistical significance was determined using one-way ANOVA following log transformation. **E**) GSL profiling of WT, one clonal line of LRRK2 R1441G KI, and one clonal line of GBA1 KO A549 cells was performed, and the percent change was measured by normalizing the average of each group to the average of WT cells. The analytes included had nominal p-values (* *p* ≤ 0.10) for genotype difference for either LRRK2 R1441G vs. WT or GBA1 KO vs. WT and were grouped based on lipid class; *n* = 3 independent experiments. White in the color scale depicts the WT reference level as 100%, red depicts an accumulation (capped at 350%), and blue depicts a reduction; *p ≤ 0.05, **p ≤ 0.01, ***p ≤ 0.001, ****p ≤ 0.0001

**Fig. 5:**
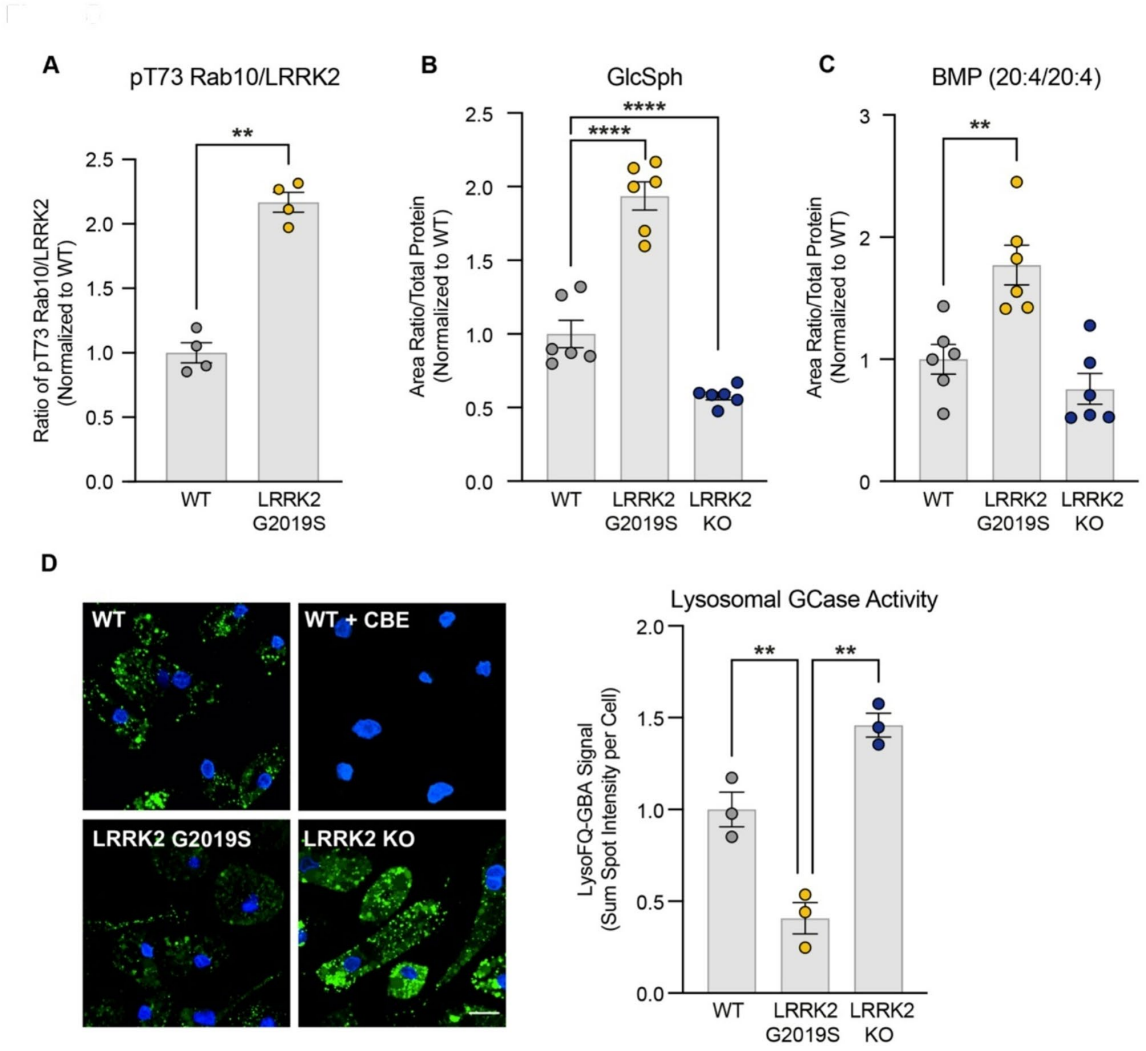
LRRK2 regulates GCase activity and levels of BMP and glucosylsphingosine in human iPSC-derived microglia. **A**) pT73 Rab10 levels and total LRRK2 were quantified from WT and LRRK2 G2019S KI iMicroglia using MSD-based assays. The ratio of pRab10/LRRK2 levels were quantified, and data are shown as mean ± SEM; *n* = 4 independent experiments, and statistical significance was determined using Student’s t-test. **B** and **C**) The levels of GlcSph and BMP (20:4/20:4) were measured using LC-MS/MS in cell lysates from WT, LRRK2 G2019S KI and LRRK2 KO iMicroglia. Data are shown as mean ± SEM; *n* = 6 independent experiments, and statistical significance was determined using one-way ANOVA and Tukey’s multiple comparison. **D**) Lysosomal GCase activity was assessed using the LysoFQ-GBA probe in WT cells, WT cells treated with the GCase inhibitor CBE, LRRK2 G2019S KI, and LRRK2 KO iMicroglia. The sum of the spot intensities per cell was quantified; data are shown as mean ± SEM; *n* = 3 independent experiments, and statistical significance was determined using one-way ANOVA and Tukey’s method for multiple comparisons. **p ≤ 0.01, ****p ≤ 0.0001

**Fig. 6:**
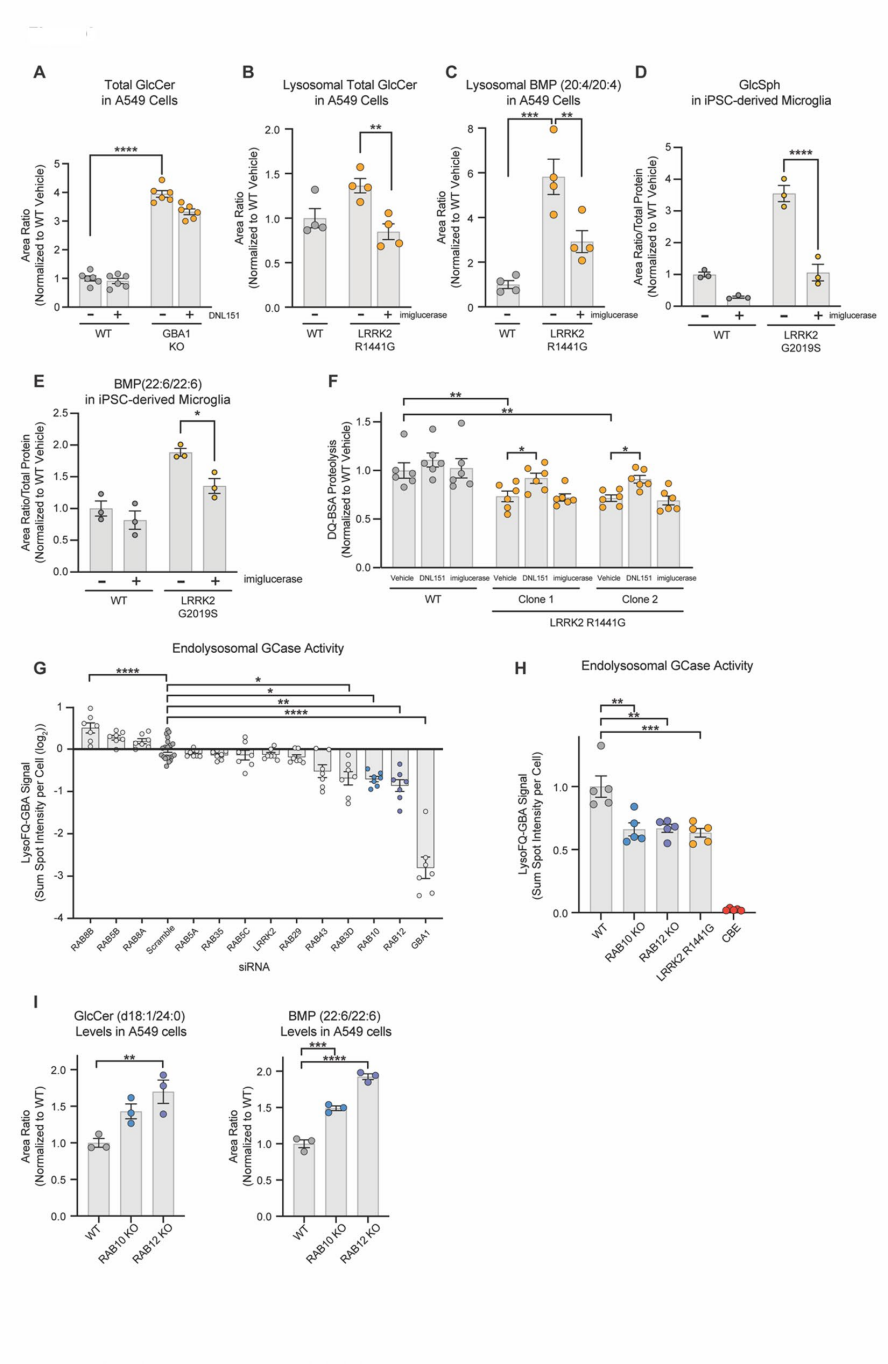
GCase is necessary and sufficient to mediate LRRK2’s effects on the levels of GCase substrates and BMP, and GCase activity is regulated by the LRRK2 substrates Rab10 and Rab12. **A**) WT and one clonal line of GBA1 KO A549 cells were treated with vehicle or DNL151 (2µM) for 72 h, and the levels of GlcCer species were measured using LC-MS/MS. The sum of all GlcCer species was measured, and data are shown as mean ± SEM; *n* = 6 independent experiments, and statistical significance was determined using one-way ANOVA following log transformation. **B** and **C**) WT and one clonal line of LRRK2 R1441G KI A549 cells were treated with vehicle or imiglucerase (2µM) for 72 h, and the levels of GlcCer and BMP (20:4/20:4) were measured using LC-MS/MS. Data are shown as mean ± SEM; *n* = 4 independent experiments, and statistical significance was determined using one-way ANOVA following log transformation. **D** and **E**) WT and LRRK2 G2019S iMicroglia were treated with vehicle or imiglucerase (1µM) for 72 h, and the levels of GlcSph and BMP (22:6/22:6) were measured using LC-MS/MS. Data are shown as mean ± SEM; *n* = 3 independent experiments, and statistical significance was determined using two-way ANOVA with Sidak’s multiple comparison test. **F**) WT and two clones of LRRK2 R1441G KI A549 cells were treated with vehicle, DNL151 (2µM), or imiglucerase (2µM) for 72 h. The sum of spot intensities of the DQ-BSA fluorescence signal was quantified per cell. DQ-BSA signal was normalized to the median within each experiment and to the average of the WT controls across experiments. Data are shown as mean ± SEM; *n* = 6 independent experiments and statistical significance was determined using two-way ANOVA with Sidak’s multiple comparison test following log transformation. **G**) siRNA KD screen of LRRK2 substrate Rabs identifies modifiers of GCase activity in A549 cells. Rab expression was transiently knocked down in WT A549 cells using transfection of pooled targeted siRNAs, and GCase activity was evaluated using the live cell LysoFQ-GBA probe. Data are shown as mean ± SEM; *n* = 7 independent experiments, and statistical significance was determined using one-way ANOVA following log transformation. **H**) Reduced GCase activity was confirmed in RAB10 KO and RAB12 KO A549 cells; Data are shown as mean ± SEM; *n* = 5 independent experiments, and statistical significance was determined using one-way ANOVA following log transformation. **I**) Levels of GlcCer(d18:1/24:0) and BMP(22:6/22:6) were measured in WT, RAB10 KO and RAB12 KO A549 cells using LCMS/MS. Data are shown as mean ± SEM; *n* = 5 independent experiments, and statistical significance was determined using one-way ANOVA following log transformation; *p ≤ 0.05, **p ≤ 0.01, ***p ≤ 0.001, ****p ≤ 0.0001

**Fig. 7:**
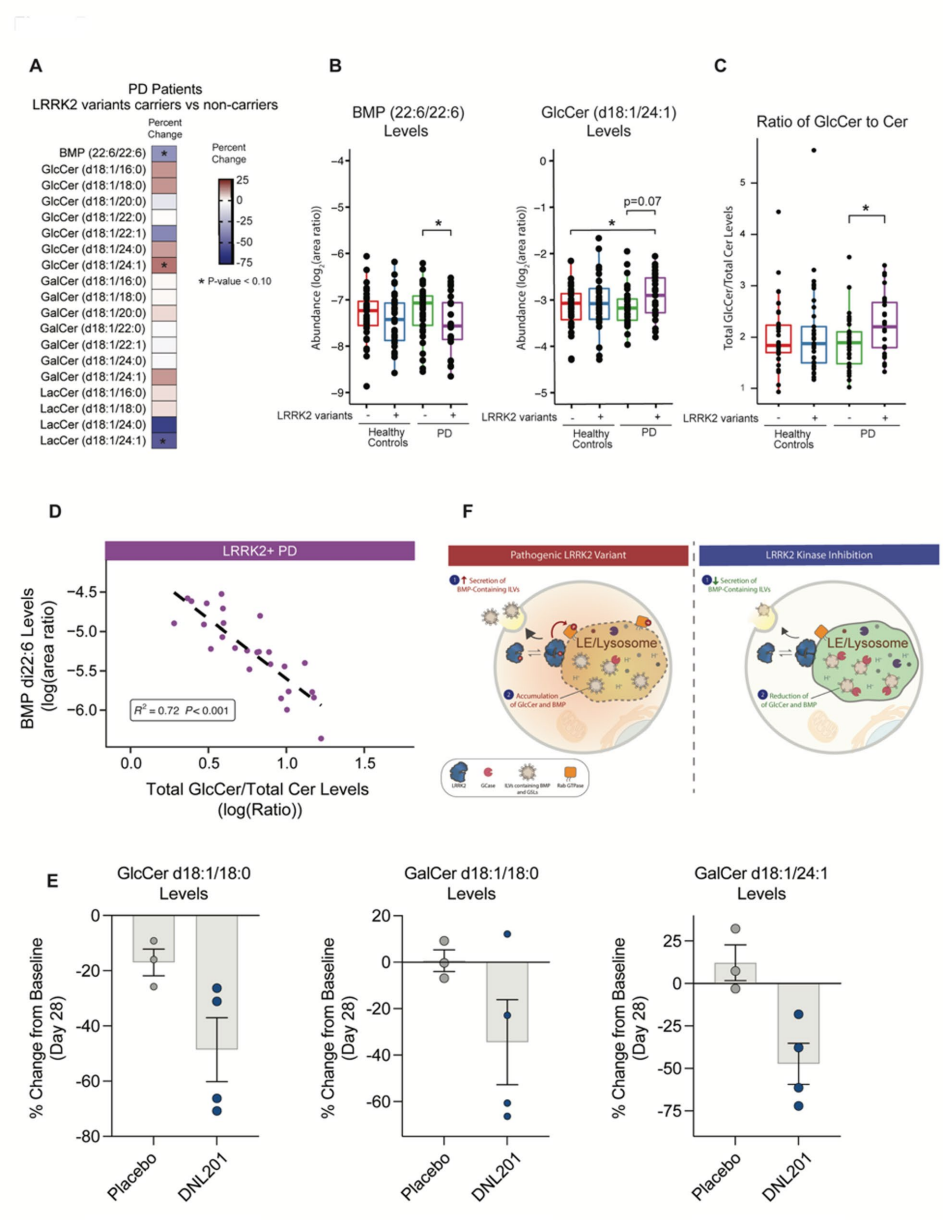
Lipidomic analysis of CSF shows alterations in GlcCer and BMP in PD patients with LRRK2 variants. Targeted analyses of CSF lipid levels were determined by LC-MS/MS in human subjects from the LCC cohort (Healthy controls without LRRK2 variants: *n* = 35; Healthy controls with LRRK2 variants: *n* = 37; PD patients without LRRK2 variants: *n* = 37; PD patients with LRRK2 variants: *n* = 26. **A**) Heatmap showing percent change in lipid abundance detected in CSF from PD patients with LRRK2 variant carriers compared to non-carriers. Percent changes and significance of effects were analyzed using robust linear model with sex and age as covariates. * unadjusted *p* ≤ 0.10. **B**) Relative abundance of BMP(22:6/22:6) and GlcCer(d18:1/24:1) levels were measured in CSF. Significance of change was analyzed by linear model with pairwise comparisons by Tukey’s honest significant difference test with significance set at unadjusted p value of 0.05. Main box and error bars depict interquartile ranges of top 75th or bottom 25th percentile and largest and smallest value with 1.5 times the interquartile ranges above and below 75th or 25th percentiles. Median 50th percentile is shown as midline within each boxplot. *p ≤ 0.05. **C**) The ratio of total GlcCer to total ceramide (Cer) levels were measured across study participants. Total GlcCer was calculated as sum of area ratios from GlcCer(d18:1/16:0), GlcCer(d18:1/18:0), GlcCer(d18:1/24:0), and GlcCer(d18:1/24:1). Total Cer was calculated as sum of area ratios from Cer lipids with identically matched acyl chain groups as GlcCer quantified. Significance of change was analyzed by linear model with pairwise comparisons by Tukey’s honest significant difference test. Ranges of values illustrated are the same as (B); *p ≤ 0.05. **D**) Pearson correlation coefficient and significance of correlations between BMP(22:6/22:6) and GlcCer(d18:1/24:1) in LRRK2 variant carriers with PD. **E**) The levels of GlcCer(d18:1/18:0), GalCer(d18:1/18:0), and GalCer(d18:1/24:1) were measured in CSF in PD subjects that carry a LRRK2 variant at baseline and following 28 days of dosing with placebo (*n* = 3) or DNL201 (*n* = 4). The percentage change from baseline was analyzed by calculating the lipid abundance change from the day 28-post-dose to pre-dose-baseline, and then normalizing the change to lipid abundance at pre-dose baseline. Data are shown as mean ± SEM. **F**) Model for mechanisms by which LRRK2 regulates BMP and GSL levels. Pathogenic variants in LRRK2 lead to an increased phosphorylation of relevant Rabs at the lysosome, impaired endolysosomal GCase activity, and reduced lysosomal proteolysis. We propose two potential mechanisms that are employed depending on the tissue examined in response to LRRK2-mediated lysosomal dysfunction: (1) in kidney, we propose that LRRK2 hyperactivity promotes increased secretion of BMP- and GSL-containing vesicles as a compensatory response to help clear accumulated lipids and proteins and (2) in other cell types, including in the brain, increased LRRK2 activity leads to an increase in intracellular GCase substrate levels, and BMP is upregulated as a compensatory response to boost GCase activity. LRRK2 kinase inhibition corrects lysosomal dysfunction and obviates the need for compensatory measures to restore lysosomal homeostasis, leading to a reduction in BMP and GSL secretion and BMP and GCase substrate accumulation in cells

## Methods

### Genotype analysis of BMP levels in urine from human subjects

We used publicly available data from PPMI to explore the relationship between PD risk and protective variants in *LRRK2* and urine BMP levels. For the genetic data, we used WGS data on PPMI subjects available through the Accelerating Medicines Partnership– Parkinson’s Disease (AMP-PD) data portal. Quality control and processing of these data has been previously described [36]. A total of 10,418 samples were included in the AMP-PD WGS data (data release 2021_v2-5release_0510), of which 1,807 were from the PPMI study. To predict ancestry of these samples, we merged the data with 1000 Genomes Phase 3 (1000G) reference samples [37]. After linkage disequilibrium pruning this merged dataset, we performed principal component analysis (PCA) and then used the first 10 principal components and 1000G super population labels to predict genetic ancestry for all samples in our dataset via the k-nearest neighbors algorithm. Because a vast majority (*n* = 1,741) of PPMI samples were predicted to be of European ancestry, we elected to restrict our downstream analyses to just this group.

Urine BMP measurements were available in PPMI for the following species of BMP: total BMP (22:6/22:6), 2,2’(22:6/22:6), and total BMP(18:1/18:1). These measurements, along with those for urinary creatinine, were generated under PPMI Project ID #145; full details on measurement methodology and data processing can be found in the methods document for this project available through PPMI. After restricting to measurements taken at the PPMI baseline visit, BMP measurements (reported in ng/mg creatinine) were available on 1,232 subjects. Of these, 1,133 samples overlapped with the 1,741 predicted European ancestry samples with available WGS data. Finally, we removed 69 samples with a case/control designation of “Other” in the available AMP-PD metadata, a classification which included prodromal subjects and those with other neurological disorders. Therefore, a final cohort of 1,069 subjects (classified as having PD or as controls only) with joint WGS and urine BMP measurements were used for SNP association testing.

### Animal care

All procedures in animals were performed with adherence to ethical regulations and protocols approved by Denali Therapeutics Institutional Animal Care and Use Committee. Mice were housed under a 12-hour light/dark cycle and had access to water and standard rodent diet (#25502, irradiated; LabDiet) ad libitum. In-diet dosing studies utilized compound-specific rodent chow outlined below.

### Mouse strains and subject details

*LRRK2*-KO (C57BL/6-Lrrk2tm1.1Mjff/J) and LRRK2 G2019S KI mice (C57BL/6-Lrrk2tm4.1Arte) mice were obtained from The Jackson Laboratory (Strain #016121) and Taconic Biosciences Inc (Model# 13940), respectively. Generally, genetically modified mice and their WT littermates were age- and sex-matched across groups for experiments. 4–6 months old *LRRK2* KO and WT mice were used to analyze BMP and GSL concentrations in urine (Figs. 1B and 2A), kidney (Figs. 1D and 2B-C). 4–6 months old *LRRK2* KO and WT mice, female only, were used for FACS-sorted brain cells (Fig. 3B-D) due to availability of the mouse colony. LRRK2 G2019S KI and WT mice were sacrificed at 5–6 months of age (young) and 18 months of age (old) to evaluate BMP and GSL concentrations in FACS-sorted brain cells (Fig. 3E and I). 5–8 months old LRRK2 G2019S KI and WT mice, treated with vehicle or MLi-2 (LRRK2 inhibitor) diet for 35 days, were used to analyze BMP and GSL concentrations in kidney (Fig. 2D, F, G) and urine (Fig. 2E).

### LRRK2 inhibitor treatment in LRRK2 G2019S KI mice

MLi-2 (MedChemExpress, Monmouth Junction, NJ) was used as a tool LRRK2 inhibitor for in vivo experiments. Grain-based, bacon-flavored rodent chow was formulated into pellets containing MLi-2 (960 mg/kg diet, estimated to be 100 mg/kg per mice) and irradiated for in-diet dosing (Bio-Serv; LabDiet; PicoLab Mouse Diet 20, 5058). Mice were provided with vehicle or MLi-2 diet ad libitum for 35 days. Food weight and mouse body weight were routinely monitored to evaluate diet consumption and animal health.

### Biofluid and tissue collection from LRRK2 mouse models

For studies without MLi-2 treatment, urine was collected over 3 consecutive days to capture animals that did not urinate in initial attempts. For MLi-2 treatment studies, urine was collected on the day of terminal tissue collection. Urine samples were snap-frozen on dry ice and transferred to − 80 °C for storage. For terminal tissue collection, mice were anesthetized with 2.5% tribromoethanol. Once deeply anesthetized, animals were transcardially perfused with ice-cold PBS using a peristaltic pump for a minimum of 3 min at a rate of 5 mL/min. After perfusion, brain was collected for subsequent FACS analysis (see section below). Kidney was removed and sub-dissected into the renal cortex and medulla. Each portion was weighed (20 ± 2 mg), collected in 1.5-mL Eppendorf tubes, frozen on dry ice, and stored at − 80 °C. Urine, renal medulla and renal cortex samples were prepared for lipid extraction, and extracted BMP and GSL species were quantified.

### MALDI-imaging mass spectrometry analysis of BMP levels in kidney and brain of LRRK2 KO mice

#### Sample Preparation

*LRRK2* KO mice and their WT littermates (5 to 6 months old) were used for MALDI-IMS experiments. Following transcardial perfusion with ice-cold PBS, brain and kidney tissues were collected and flash frozen on aluminum foil that was slowly lowered into liquid nitrogen for approximately 10 s. Frozen tissue was stored at -80 °C until ready for use. Prior to sectioning, the tissues were removed from the − 80 °C freezer and placed in the cryostat chamber to equilibrate to -20 °C. Brain and kidney tissues were cut on a cryostat (Leica Biosystems) into 12 μm thick sections and thaw-mounted onto indium-tix oxide (ITO) coated glass slides (Delta Technologies). Additional sections were obtained for H&E staining. After staining, digital micrographs were obtained via a slide scanner (Leica Biosystems).

#### Matrix application

The ITO-coated slides were coated with 1,5-diaminonaphthalene (DAN) MALDI matrix via sublimation [38, 39]. For kidney, a custom glass sublimation apparatus was used. Briefly, 100 mg of recrystallized DAN was placed in the bottom of a glass sublimation apparatus (Chemglass Life Sciences). The apparatus was placed on a metal heating block set to 130 °C and DAN was sublimated onto the tissue surface for 4 min at a pressure of less than 25 mTorr. Approximately 2.8 mg of DAN was applied to the ITO slide, determined by weighing the slide before and after matrix application. A home-built sublimation device was used for the brain sections. Briefly, approximately 19 mg of recrystallized DAN was dissolved in 2 mL of acetone and deposited on the bottom of the device. Heat was applied for 10 min at > 130 °C and all DAN was sublimated. The coated plates were then subjected to mass spectrometer analysis.

#### Imaging mass spectrometry

The tissue sections were imaged on a Solarix 15T FT-ICR MS (Bruker Daltonics), equipped with a SmartBeam II 2 kHz frequency tripled Nd: YAG laser (355 nm). Images were acquired at 125 μm (kidney) or 100 μm (brain) step size in negative ion mode. Each pixel is the average of 1500 laser shots using the small laser focus setting and random-walking within the pixel. The mass spectrometer was externally calibrated with a series of red phosphorus clusters. Data were collected from m/z 345–1,000. Images were generated using FlexImaging 3.0 (Bruker Daltonics). BMP was identified by accurate mass, with the mass accuracies typically better than 1 ppm. Brain ROIs were selected corresponding to the cortex, midbrain, hippocampus, and striatum (Supplementary Fig. 4C). Average spectra for the ROIs were exported from FlexImaging as.csv files and imported into mMass [38, 39]. Spectra for all four ROIs were overlaid to directly compare peak intensities. The resulting graphs for m/z 865.502 (corresponding to BMP 22:6/22:6) and m/z 834.530 (corresponding to PS 40:6) are shown in (Supplementary Fig. 4E and 4F).

### FACS-based analysis of BMP and glycosphingolipid levels in mouse CNS cells

Mice were perfused with PBS, and whole brains dissected, and processed into a single cell suspension according to the manufacturers’ protocol using the adult brain dissociation kit (Miltenyi Biotec 130-107-677). Cells were Fc blocked (Biolegend #101320, 1:100) and stained for flow cytometric analysis with Fixable Viability Stain BV510 (BD Biosciences #564406, 1:100) to exclude dead cells, CD11b-BV421 (BD Biosciences 562605, 1:100), CD31-PerCP Cy5.5 (BD Biosciences #562861, 1:100), O1-488 (Thermo/eBio #14-6506-82, 1:50), Thy1-PE (R&D #FAB7335P, 1:100), and EAAT2-633 (Alomone #AGC-022-FR, 1:60). Cells were washed with PBS/1% BSA and strained through a 100 μm filter before sorting CD11b + microglia, EAAT2 + astrocytes, and Thy1 + neurons on a FACS Aria III (BD Biosciences) with a 100 μm nozzle. In order to achieve pure populations of astrocytes, microglia, and neurons negative gates were set to remove O1 + and CD31 + cells which are predominantly oligodendrocytes and endothelial cells respectively. The whole procedures including single cell suspension, staining, and sorting were kept at 4 C. Sorted cells were directly collected into methanol extraction buffer for LC-MS/MS analysis (described below).

### LC-MS based analysis of lipids

#### Sample Preparation

FACS-sorted brain cells: 100,000 cells were sorted directly into tubes containing 800 µL of LC-MS grade methanol containing 2 µL of internal standard mix. These samples were vortex for 5 min, and centrifuged at 21,000 x g for 10 min at 4 °C. The supernatants were transferred to a new glass vial, and half of the sample was aliquoted for GlcCer/GalCer analysis, while half of the sample was used for other lipid panel analysis. The samples were dried down under a constant stream of N_2_, and stored at -80 °C until analysis.

Kidney and brain tissues: Approximately 20 mg of kidney samples were placed in Safe-Lock Eppendorf tube (Eppendorf Cat#022600044) containing 5 mm stainless steel beads (QIAGEN Cat#69989). To these tubes, 400 µL of LC-MS grade methanol containing 2 µL internal standard mix solution was added and homogenized for 30 s at 25 HZ at 4 °C with Tissuelyser. Homogenized samples were centrifuged at 21,000 x g for 20 min at 4 °C. Cleared methanolic supernatants were transferred into new Eppendorf vials and kept in -20 °C for 1 h. Samples were centrifuged at 21,000 g for 10 min at 4 °C and supernatants were aliquoted into 96 well plates with glass inserts and dried under a constant stream of N_2_. Dried samples were stored in -80 °C until analysis.

Genotype and drug-treatment analysis in cell lysates: A549 cells were seeded into standard 6-well cell culture plates at a density of 90 K cells/well. For iMicroglia, cells were seeded at 20 K cells/well into CellCarrier-96 Ultra Microplates (Perkin Elmer #6055302). At the time of harvest, cell media was aspirated and cells were washed with ice-cold 0.9% NaCl “Normal Saline”. Cells were then quickly extracted into 400 µL per well of ice-cold extraction buffer (9:1; LCMS-grade MeOH: H2O) plus 2µL of internal standards. Cell extracts were scrapped off the well then transferred into a clean Eppendorf tube. Extracts were then shaken at 2000 r.p.m. for 20 min at 4 °C, then centrifuged at 21,000 x g at 4 °C for 5 min to pellet insoluble material. Next, 100 µL of the cleared methanolic supernatant was transferred into glass vials or plates with glass inserts. These samples were dried under a stream of N_2_ gas for 4 h and stored in -80 °C until analysis. To assess the effect of recombinant GCase addition to lipid levels in cells, cells were supplemented with imiglucerase (Sanofi) at a final concentration of 2 µM in water. A549 cells were treated with imiglucerase for 96 h with full replacement/exchange of medium at 48 h. For iMicroglia plated in NGD + media, cells were treated with 1µM imiglucerase for 72 h. Cells were then processed for downstream analysis as described above.

Exosomes from cell culture media: WT or LRRK2 G2019S iMG were grown in NGD + media for 72 h. Supernatant media was collected and centrifuged at 2500 g for 10 min. to cell debris. Supernatant was then transferred for exosome isolation according to the EVTRAP method previously described [40]. Following elution, samples were analyzed by mass spectrometry.

Urine and CSF sample extraction: Samples were centrifuged at 1000 x g at 4 °C to remove particulates. 20 µL of urine and 10 µL of CSF samples were transferred into a 2 mL Safe-lock Eppendorf tubes. To these samples, 200 µL of LC-MS grade methanol containing 2 µL of internal standard mix were added and vortexed for 5 min. Samples were centrifuged at 21,000 x g for 4 °C and stored at 20 °C for 1 h. Following this step, samples were centrifuged once again at 21,000 x g for 20 min at 4 °C. Resultant supernatants were transferred to 96 well plates with glass inserts and dried under constant stream of N_2_. Dried samples were stored at -80 °C until analysis.

#### Sample Preparation for glucosylceramide and Galactosylceramide measurements

For analysis and separation of glucosylceramides and galactosylceramides, 50 µL of above extracts were dried under constant stream of N_2_ for 4 h and resuspended in 200 µL of 92.5/5/2.5 LC-MS grade acetonitrile/isopropanol/water fortified with 5 mM ammonium formate and 0.5% formic acid.

#### LCMS targeted analysis of lipids

Lipid analyses were performed by liquid chromatography (UHPLC Nexera X2, UHPLC ExionLC) coupled to electrospray mass spectrometry (QTRAP 6500+). For each analysis, 5 µL of sample was injected on a BEH C18 1.7 μm, 2.1 × 1 00 mm column (Waters) using a flow rate of 0.25 mL/min at 55 °C. For positive ionization mode, mobile phase A consisted of 60:40 acetonitrile/water (v/v) with 10 mM ammonium formate + 0.1% formic acid; mobile phase B consisted of 90:10 isopropyl alcohol/acetonitrile (v/v) with 10 mM ammonium formate + 0.1% formic acid. For negative ionization mode, mobile phase A consisted of 60:40 acetonitrile/water (v/v) with 10 mM ammonium acetate + 0.1% acetic acid; mobile phase B consisted of 90:10 isopropyl alcohol/acetonitrile (v/v) with 10 mM ammonium acetate + 0.1% acetic acid. The gradient was programmed as follows: 0.0–8.0 min from 45% B to 99% B, 8.0–9.0 min at 99% B, 9.0–9.1 min to 45% B, and 9.1–10.0 min at 45% B. Electrospray ionization was performed in positive or negative ion mode. For the QTRAP 6500+, we applied the following settings: curtain gas at 30 psi (negative mode) and curtain gas at 40 psi (positive mode); collision gas was set at medium; ion spray voltage at 5500 V (positive mode) or -4500 V (negative mode); temperature at 250 °C (positive mode) or 600 °C (negative mode); ion source Gas 1 at 55 psi; ion source Gas 2 at 60 psi; entrance potential at 10 V (positive mode) or -10 V (negative mode); and collision cell exit potential at 12.5 V (positive mode) or -15.0 V (negative mode). Data acquisition was performed in multiple reaction monitoring mode (MRM) with the collision energy (CE) values reported in Tables 1 and 2. Lipids were quantified using a mixture of non-endogenous internal standards as reported in Tables 1 and 2. Quantification was performed using MultiQuant 3.02.

#### LCMS targeted analysis of GlcCer and GalCer

Glucosylceramide, galactosylceramide and glucosylsphingosine analyses were performed by liquid chromatography (UHPLC Nexera X2, UHPLC ExionLC) coupled to electrospray mass spectrometry (TQ 6495 C). For each analysis, 5 µL of sample was injected on a HALO HILIC 2.0 μm, 3.0 × 150 mm column (Advanced Materials Technology, PN 91813-701) using a flow rate of 0.48mL/min at 45 °C. Mobile phase A consisted of 92.5/5/2.5 ACN/IPA/H2O with 5 mM ammonium formate and 0.5% formic acid. Mobile phase B consisted of 92.5/5/2.5 H2O/IPA/ACN with 5 mM ammonium formate and 0.5% formic acid. The gradient was programmed as follows: 0.0–2 min at 100% B, 2.1 min at 95% B, 4.5 min at 85% B, hold to 6.0 min at 85% B, drop to 0% B at 6.1 min and hold to 7.0 min, ramp back to 100% at 7.1 min and hold to 8.5 min. Electrospray ionization was performed in positive mode. Agilent TQ 6495 C was operated with the following settings: gas temp at 180 °C; gas flow 17 L/min; nebulizer 35 psi; sheath gas temp 350 °C; sheath gas flow 10 L/min; capillary 3500 V; nozzle voltage 500 V. Glucosylceramide and galactosylceramide species were identified based on their retention times and MRM properties of commercially available reference standards (Avanti Polar Lipids, Birmingham, AL, USA). Quantification was performed using Skyline (v19.1; University of Washington). Table 3 shows specific analytes and internal standards used in this assay.

**Table 1:** Lipidomics in positive mode parameters

Name	Internal Standard	Q1 m/z	Q3 m/z	CE (V)
1-O-Palmitoyl-Cer(d18:1/18:0)	Cer(d18:1/16:0(d7))	786.8	502.5	35
24-Hydroxycholesterol	24-Hydroxcholesterol-d7	385.3	367.3	30
24-Hydroxycholesterol(d7)	IS	392.3	367.3	30
3-O-SulfoLacCer(d18:1/18:0)	LacCer(d18:1/17:0)	970.8	548.5	61
4-beta-Hydroxycholesterol	Cholesterol(d7)	420.3	385.3	15
7-keto-Cholesterol	Cholesterol(d7)	401.3	383.3	15
CE HETE	CE(18:1(d7))	706.6	369.2	25
CE HODE	CE(18:1(d7))	682.6	369.2	25
CE HpODE	CE(18:1(d7))	698.6	369.2	25
CE oxoHETE	CE(18:1(d7))	704.6	369.2	25
CE oxoODE	CE(18:1(d7))	680.6	369.2	25
CE(16:1)	CE(18:1(d7))	640.6	369.3	26
CE(18:1(d7))	IS	675.2	369.4	26
CE(18:1)	CE(18:1(d7))	668.6	369.3	26
CE(18:2)	CE(18:1(d7))	666.6	369.3	26
CE(20:4)	CE(18:1(d7))	690.6	369.3	26
CE(20:5)	CE(18:1(d7))	688.6	369.3	26
CE(22:6)	CE(18:1(d7))	714.6	369.3	26
Cer(d18:0/16:0)	Cer(d18:1/16:0(d7))	540.6	284.3	40
Cer(d18:0/18:0)	Cer(d18:1/16:0(d7))	568.7	284.3	40
Cer(d18:0/24:0)	Cer(d18:1/16:0(d7))	652.9	284.3	40
Cer(d18:0/24:1)	Cer(d18:1/16:0(d7))	650.9	284.4	40
Cer(d18:1/16:0(d7))	IS	545.5	271.4	40
Cer(d18:1/16:0)	Cer(d18:1/16:0(d7))	538.5	264.3	40
Cer(d18:1/18:0)	Cer(d18:1/16:0(d7))	566.6	264.3	40
Cer(d18:1/24:0)	Cer(d18:1/16:0(d7))	650.6	264.3	40
Cer(d18:1/24:1)	Cer(d18:1/16:0(d7))	648.6	264.3	40
Cholesterol	Cholesterol(d7)	369.3	369.3	10
Cholesterol(d7)	IS	376.2	376.2	10
Cholesteryl hexoside	CE(18:1(d7))	566.6	369.3	17
Coenzyme Q10	TG(15:0/18:1(d7)/15:0)	863.3	197.2	35
DG(15:0/18:1(d7))	IS	605.6	346.5	30
DG(16:0_18:1)	DG(15:0/18:1(d7))	612.4	313.3	30
DG(16:0_20:4)	DG(15:0/18:1(d7))	634.5	313.3	30
DG(18:0_18:1)	DG(15:0/18:1(d7))	640.4	341.3	30
DG(18:0_20:4)	DG(15:0/18:1(d7))	662.5	341.3	30
DG(18:0_22:6)	DG(15:0/18:1(d7))	686.6	341.3	30
DG(18:1_20:4)	DG(15:0/18:1(d7))	660.5	339.3	30
DG(18:1/18:1)	DG(15:0/18:1(d7))	638.4	339.3	30
GB3(d18:1/16:0)	GB3(d18:1/18:0(d3))	1025	520.5	40
GB3(d18:1/18:0(d3))	IS	1056	551.6	40
GB3(d18:1/18:0)	GB3(d18:1/18:0(d3))	1053	548.6	40
GB3(d18:1/24:0)	GB3(d18:1/18:0(d3))	1137	632.6	40
GB3(d18:1/24:1)	GB3(d18:1/18:0(d3))	1135	630.6	40
GlcCer(d18:1(d5)/18:0)	IS	733.6	269.3	45
GlcCer(d18:1/16:0(d3))	IS	703.7	264.3	51
Glucosylsphingosine(d5)	IS	467.2	269.3	16
HexCer(d18:1/12:0)	GlcCer(d18:1(d5)/18:0)	644.5	264.3	40
HexCer(d18:1/16:0)	GlcCer(d18:1(d5)/18:0)	700.6	264.3	40
HexCer(d18:1/18:0)	GlcCer(d18:1(d5)/18:0)	728.6	264.3	40
HexCer(d18:1/22:0)	GlcCer(d18:1(d5)/18:0)	784.7	264.4	40
HexCer(d18:1/24:0)	GlcCer(d18:1(d5)/18:0)	812.7	264.3	40
HexCer(d18:1/24:1)	GlcCer(d18:1(d5)/18:0)	810.7	264.3	40
Hexosylsphingosine	Glucosylsphingosine(d5)	462.3	264.2	16
LacCer(d18:1/16:0)	LacCer(d18:1/17:0)	862.6	264.3	40
LacCer(d18:1/17:0)	IS	876.6	264.3	40
LacCer(d18:1/18:0)	LacCer(d18:1/17:0)	890.7	264.3	40
LacCer(d18:1/24:0)	LacCer(d18:1/17:0)	974.8	264.3	40
LacCer(d18:1/24:1)	LacCer(d18:1/17:0)	972.7	264.3	40
Lactosylsphingosine	Glucosylsphingosine(d5)	624.4	264.3	16
LPC(16:0)	LPC(18:1(d7))	496.3	184.1	40
LPC(16:1)	LPC(18:1(d7))	494.5	184.1	40
LPC(18:0)	LPC(18:1(d7))	524.3	184.1	40
LPC(18:1(d7))	IS	529.3	184.1	40
LPC(18:1)	LPC(18:1(d7))	522.3	184.1	40
LPC(20:4)	LPC(18:1(d7))	544.3	184.1	40
LPC(22:6)	LPC(18:1(d7))	568.3	184.1	40
LPC(24:0)	LPC(18:1(d7))	608.5	184.1	40
LPC(24:1)	LPC(18:1(d7))	606.5	184.1	40
LPC(26:0)	LPC(18:1(d7))	636.5	104.1	40
LPC(26:1)	LPC(18:1(d7))	634.5	104.1	40
lyso-GB3	lyso-GB3-d7	786.6	264.3	46
lyso-GB3-d7	IS	793.5	271.3	46
lyso-GB4	GB3(d18:1/18:0(d3))	990.6	264.3	52
MG(16:0)	MG(18:1(d7))	348.3	239.3	22
MG(16:1)	MG(18:1(d7))	346.3	237.3	22
MG(18:0)	MG(18:1(d7))	376.3	267.3	22
MG(18:1(d7))	IS	381.3	272.5	22
MG(18:1)	MG(18:1(d7))	374.3	265.3	22
MG(20:4)	MG(18:1(d7))	396.3	287.3	22
N-Oleoylethanolamine	LPC(18:1(d7))	326.3	62.1	23
N-Palmitoyl-O-phosphocholineserine	LPC(18:1(d7))	509.5	184.1	40
Palmitoylethanolamine	LPC(18:1(d7))	300.3	62.1	23
PC(15:0/18:1(d7))	IS	754.6	184.1	40
PC(16:0/5:0(CHO))	PC(15:0/18:1(d7))	594.5	184.1	40
PC(16:0/9:0(CHO))	PC(15:0/18:1(d7))	650.4	184.1	40
PC(16:0/9:0(COOH))	PC(15:0/18:1(d7))	666.4	184.1	40
PC(18:0/20:4(OH[S]))	PC(15:0/18:1(d7))	826.6	184.1	40
PC(18:0/20:4(OOH[S]))	PC(15:0/18:1(d7))	842.6	184.1	40
PC(34:1)	PC(15:0/18:1(d7))	760.6	184.1	40
PC(36:1)	PC(15:0/18:1(d7))	788.6	184.1	40
PC(36:2)	PC(15:0/18:1(d7))	786.6	184.1	40
PC(36:4)	PC(15:0/18:1(d7))	782.6	184.1	40
PC(38:4)	PC(15:0/18:1(d7))	810.6	184.1	40
PC(38:6)	PC(15:0/18:1(d7))	806.6	184.1	40
PC(40:5)	PC(15:0/18:1(d7))	836.6	184.1	40
PC(40:6)	PC(15:0/18:1(d7))	834.6	184.1	40
PC(O-16:0/0:0)	LPC(18:1(d7))	482.3	184.1	40
PC(O-16:0/2:0)	LPC(18:1(d7))	524.3	184.2	40
PC(O-18:0/2:0)	LPC(18:1(d7))	552.5	184.1	40
PE(15:0/18:1(d7))	IS	711.6	570.5	40
PE(18:0/20:4(OH[S]))	PE(15:0/18:1(d7))	784.5	643.4	40
PE(18:0/20:4(OOH[S]))	PE(15:0/18:1(d7))	800.5	659.4	40
PE(34:1)	PE(15:0/18:1(d7))	718.6	577.6	40
PE(36:1)	PE(15:0/18:1(d7))	746.6	605.5	40
PE(36:2)	PE(15:0/18:1(d7))	744.6	603.5	40
PE(36:4)	PE(15:0/18:1(d7))	740.6	599.5	40
PE(38:4)	PE(15:0/18:1(d7))	768.6	627.5	40
PE(38:5)	PE(15:0/18:1(d7))	766.6	625.5	40
PE(38:6)	PE(15:0/18:1(d7))	764.6	623.5	40
PE(40:4)	PE(15:0/18:1(d7))	796.6	655.5	40
PE(40:5)	PE(15:0/18:1(d7))	794.6	635.5	40
PE(40:6)	PE(15:0/18:1(d7))	792.6	651.5	40
PE(40:7)	PE(15:0/18:1(d7))	790.6	649.5	40
Sitosteryl hexoside	CE(18:1(d7))	594.6	397.4	17
SM(d18:1(d9)/18:1)	IS	738.7	184.1	40
SM(d18:1/16:0)	SM(d18:1(d9)/18:1)	703.6	184.1	40
SM(d18:1/18:0)	SM(d18:1(d9)/18:1)	731.6	184.1	40
SM(d18:1/24:0)	SM(d18:1(d9)/18:1)	815.7	184.1	40
SM(d18:1/24:1)	SM(d18:1(d9)/18:1)	813.7	184.1	40
Sphinganine	Sphingosine(d17:1)	302.2	284.3	20
Sphinganine 1-phosphate	Sphingosine 1-phosphate-d7	382.3	284.3	18
Sphingosine	Sphingosine(d17:1)	300.2	264.3	20
Sphingosine 1-phosphate	Sphingosine 1-phosphate-d7	380.3	264.3	25
Sphingosine 1-phosphate-d7	IS	387.3	271.3	25
Sphingosine 1-phosphocholine	LPC(18:1(d7))	465.5	184.1	40
Sphingosine(d17:1)	IS	286.2	250.3	20
TG(15:0/18:1(d7)/15:0)	IS	829.8	570.8	40
TG(18:0_36:2)	TG(15:0/18:1(d7)/15:0)	904.7	603.4	40
TG(18:1_34:2)	TG(15:0/18:1(d7)/15:0)	874.7	575.4	40
TG(18:1_34:3)	TG(15:0/18:1(d7)/15:0)	872.7	573.4	40
TG(20:4_32:1)	TG(15:0/18:1(d7)/15:0)	870.6	549.3	40
TG(20:4_34:2)	TG(15:0/18:1(d7)/15:0)	896.6	575.3	40
TG(20:4_34:3)	TG(15:0/18:1(d7)/15:0)	894.6	573.3	40
TG(20:4_36:0)	TG(15:0/18:1(d7)/15:0)	928.8	607.5	40
TG(20:4_36:2)	TG(15:0/18:1(d7)/15:0)	924.7	603.4	40
TG(20:4_36:3)	TG(15:0/18:1(d7)/15:0)	922.7	601.4	40
TG(22:6_36:2)	TG(15:0/18:1(d7)/15:0)	948.7	603.4	40
TG(22:6_38:1)	TG(15:0/18:1(d7)/15:0)	978.7	633.4	40
TG(22:6_38:2)	TG(15:0/18:1(d7)/15:0)	976.7	631.4	40

**Table 2:** Lipidomics in negative mode parameters

Name	Internal Standard	Q1 m/z	Q3 m/z	CE (V)
(3-O-sulfo)GalCer(d18:1/16:0)	(3-O-sulfo)GalCer(d18:1/18:0(d3))	778.5	97	-150
(3-O-sulfo)GalCer(d18:1/18:0(2OH)	(3-O-sulfo)GalCer(d18:1/18:0(d3))	822.6	97	-150
(3-O-sulfo)GalCer(d18:1/18:0(d3))	IS	809.6	97	-150
(3-O-sulfo)GalCer(d18:1/18:0)	(3-O-sulfo)GalCer(d18:1/18:0(d3))	806.6	97	-150
(3-O-sulfo)GalCer(d18:1/24:0(2OH))	(3-O-sulfo)GalCer(d18:1/18:0(d3))	906.7	97	-150
(3-O-sulfo)GalCer(d18:1/24:0)	(3-O-sulfo)GalCer(d18:1/18:0(d3))	890.7	97	-150
(3-O-sulfo)GalCer(d18:1/24:1(2OH))	(3-O-sulfo)GalCer(d18:1/18:0(d3))	904.7	97	-150
(3-O-sulfo)GalCer(d18:1/24:1)	(3-O-sulfo)GalCer(d18:1/18:0(d3))	888.7	97	-150
Arachidonic acid	Arachidonic acid-d8	303.2	303.2	-10
Arachidonic acid_MRM	Arachidonic acid-d8_MRM	303.2	259.1	-19
Arachidonic acid-d8	IS	311.3	311.3	-10
Arachidonic acid-d8_MRM	IS	311.3	267.1	-19
BMP(14:0/14:0)	IS	665.3	227.2	-50
BMP(16:0_18:1)	BMP(14:0/14:0)	747.5	255.4	-50
BMP(16:0_20:4)	BMP(14:0/14:0)	769.5	255.4	-50
BMP(16:0_22:6)	BMP(14:0/14:0)	795.5	255.4	-50
BMP(16:1/16:1)	BMP(14:0/14:0)	717.5	253.1	-50
BMP(18:0_18:1)	BMP(14:0/14:0)	775.5	281.4	-50
BMP(18:0_20:4)	BMP(14:0/14:0)	797.5	283.4	-50
BMP(18:0_22:6)	BMP(14:0/14:0)	823.5	283.4	-50
BMP(18:1/18:1)	BMP(14:0/14:0)	773.5	281.3	-50
BMP(20:4/20:4)	BMP(14:0/14:0)	817.5	303.3	-50
BMP(22:6/22:6)	BMP(14:0/14:0)	865.5	327.3	-50
Cholesterol sulfate	(3-O-sulfo)GalCer(d18:1/18:0(d3))	465.3	96.7	-80
CL(14:0/14:0/14:0/14:0)	IS	619.5	227.2	-50
CL(72:6/18:2)	CL(14:0/14:0/14:0/14:0)	725.7	279.2	-50
CL(72:7/18:2)	CL(14:0/14:0/14:0/14:0)	724.7	279.2	-50
CL(72:8 − 2(OOH)/18:2)	CL(14:0/14:0/14:0/14:0)	755.7	279.2	-50
CL(72:8/18:2)	CL(14:0/14:0/14:0/14:0)	723.7	279.3	-50
CL(74:9/18:2)	CL(14:0/14:0/14:0/14:0)	736.7	279.2	-50
DHA	Arachidonic acid-d8_MRM	327.2	229.1	-19
EPA	Arachidonic acid-d8_MRM	301.3	257.1	-19
FAHFA(18:1/9-O-18:0)	PG(15:0/18:1(d7))	563.6	281	-50
GD1a/b(d36:1)	GM3(d18:1/18:0(d5))	917.5	290.1	-65
GD3(d34:1)	GM3(d18:1/18:0(d5))	720.9	290.1	-65
GD3(d36:1)	GM3(d18:1/18:0(d5))	734.9	290.1	-65
GM3(d18:1/18:0(d5))	IS	1184.8	290.1	-65
GM3(d34:1)	GM3(d18:1/18:0(d5))	1151.7	290.1	-65
GM3(d36:1)	GM3(d18:1/18:0(d5))	1179.8	290.1	-65
GQ1b(d36:1)	GM3(d18:1/18:0(d5))	1208.6	290.1	-65
Hemi-BMP(14:0/14:0)_14:0	IS	875.5	227.3	-50
Hemi-BMP(18:1/18:1)_16:0	Hemi-BMP(14:0/14:0)_14:0	1011.7	281.3	-50
Hemi-BMP(18:1/18:1)_18:0	Hemi-BMP(14:0/14:0)_14:0	1039.7	281.3	-50
Hemi-BMP(18:1/18:1)_18:1	Hemi-BMP(14:0/14:0)_14:0	1037.7	281.3	-50
Hemi-BMP(20:4/20:4)_16:0	Hemi-BMP(14:0/14:0)_14:0	1056.8	303.3	-50
Hemi-BMP(20:4/20:4)_18:0	Hemi-BMP(14:0/14:0)_14:0	1185.8	303.3	-50
Hemi-BMP(20:4/20:4)_18:1	Hemi-BMP(14:0/14:0)_14:0	1183.8	303.3	-50
Hemi-BMP(20:4/20:4)_20:4	Hemi-BMP(14:0/14:0)_14:0	1104.8	303.3	-50
Hemi-BMP(22:6/22:6)_16:0	Hemi-BMP(14:0/14:0)_14:0	1103.7	327.3	-50
Hemi-BMP(22:6/22:6)_18:0	Hemi-BMP(14:0/14:0)_14:0	1131.7	327.3	-50
Hemi-BMP(22:6/22:6)_18:1	Hemi-BMP(14:0/14:0)_14:0	1129.7	327.3	-50
Hemi-BMP(22:6/22:6)_22:6	Hemi-BMP(14:0/14:0)_14:0	1175.7	327.3	-50
Linoleic acid	Arachidonic acid-d8	279.2	279.2	-38
Linolenic acid	Arachidonic acid-d8	277.2	277.2	-10
LPE(16:0)	LPE(18:1(d7))	452.2	255.3	-50
LPE(18:0)	LPE(18:1(d7))	480.31	283.3	-50
LPE(18:1(d7))	IS	485.3	288.3	-50
LPG(16:0)	LPE(18:1(d7))	483.3	255.3	-50
LPG(18:0)	LPE(18:1(d7))	511.3	283.3	-50
LPG(18:1)	LPE(18:1(d7))	509.3	281.3	-50
LPG(20:4)	LPE(18:1(d7))	531.3	303.3	-50
LPG(22:6)	LPE(18:1(d7))	555.3	327.3	-50
LPI(16:0)	LPE(18:1(d7))	571.3	241.1	-50
LPI(18:0)	LPE(18:1(d7))	599.3	241.1	-50
Oleic acid	Arachidonic acid-d8	281.2	281.2	-10
PA(15:0/18:1(d7))	IS	666.52	241.3	-50
PA(16:0_18:1)	PA(15:0/18:1(d7))	673.5	255.3	-50
PA(18:0_18:1)	PA(15:0/18:1(d7))	701.5	283.3	-50
PA(18:0_20:4)	PA(15:0/18:1(d7))	723.5	283.3	-50
PA(18:0_22:6)	PA(15:0/18:1(d7))	747.5	283.3	-50
PA(18:1/18:1)	PA(15:0/18:1(d7))	699.5	281.3	-50
Palmitic acid	Arachidonic acid-d8	255.1	255.1	-10
Palmitoleic acid	Arachidonic acid-d8	253.1	253.1	-10
PE(15:0/18:1(d7))	IS	709.6	241.3	-50
PE(O-16:0/20:4)	PE(15:0/18:1(d7))	724.5	303.2	-50
PE(O-16:0/22:6)	PE(15:0/18:1(d7))	748.5	327.2	-50
PE(O-18:0/20:4)	PE(15:0/18:1(d7))	752.6	303.2	-50
PE(O-18:0/22:6)	PE(15:0/18:1(d7))	776.6	327.2	-50
PE(P-16:0/20:4)	PE(15:0/18:1(d7))	722.6	303.3	-50
PE(P-16:0/20:5)	PE(15:0/18:1(d7))	720.6	301.3	-50
PE(P-16:0/22:4)	PE(15:0/18:1(d7))	750.6	331.3	-50
PE(P-16:0/22:6)	PE(15:0/18:1(d7))	746.6	327.3	-50
PE(P-18:0/18:1)	PE(15:0/18:1(d7))	728.6	281.3	-50
PE(P-18:0/18:2)	PE(15:0/18:1(d7))	726.6	279.2	-50
PE(P-18:0/20:4)	PE(15:0/18:1(d7))	750.6	303.3	-50
PE(P-18:0/20:5)	PE(15:0/18:1(d7))	748.6	301.3	-50
PE(P-18:0/22:6)	PE(15:0/18:1(d7))	774.6	327.3	-50
PE(P-18:1/20:4)	PE(15:0/18:1(d7))	748.5	303.3	-50
PE(P-18:1/22:6)	PE(15:0/18:1(d7))	772.5	327.3	-50
PEth(16:0_18:1)	PE(15:0/18:1(d7))	772.5	255.1	-50
PEth(18:1/18:1)	PE(15:0/18:1(d7))	773.6	281.2	-50
PG(15:0/18:1(d7))	IS	740.55	241.3	-50
PG(16:0_18:1)	PG(15:0/18:1(d7))	747.5	255.3	-50
PG(16:0_20:4)	PG(15:0/18:1(d7))	769.5	255.3	-50
PG(16:0_22:6)	PG(15:0/18:1(d7))	795.5	255.3	-50
PG(18:0_18:1)	PG(15:0/18:1(d7))	775.5	281.3	-50
PG(18:0_20:4)	PG(15:0/18:1(d7))	797.5	283.3	-50
PG(18:0_22:6)	PG(15:0/18:1(d7))	823.5	283.3	-50
PG(18:1/18:1)	PG(15:0/18:1(d7))	773.4	281.4	-50
PI(15:0/18:1(d7))	IS	828.6	241.3	-50
PI(16:0_18:1)	PI(15:0/18:1(d7))	835.6	255.3	-50
PI(16:0_20:4)	PI(15:0/18:1(d7))	857.6	255.3	-50
PI(16:0_22:6)	PI(15:0/18:1(d7))	881.6	255.3	-50
PI(18:0_18:1)	PI(15:0/18:1(d7))	863.6	283.3	-50
PI(18:0_20:4)	PI(15:0/18:1(d7))	885.6	283.3	-50
PI(18:0_22:6)	PI(15:0/18:1(d7))	909.6	283.3	-50
PI(18:1/18:1)	PI(15:0/18:1(d7))	861.6	281.3	-50
PI(20:4/20:4)	PI(15:0/18:1(d7))	905.6	303.3	-50
PS(15:0/18:1(d7))	IS	753.55	241.3	-50
PS(16:0_18:1)	PS(15:0/18:1(d7))	760.6	255.3	-50
PS(16:0_20:4)	PS(15:0/18:1(d7))	782.6	255.3	-50
PS(16:0_22:6)	PS(15:0/18:1(d7))	806.6	255.3	-50
PS(18:0_18:1)	PS(15:0/18:1(d7))	788.6	283.3	-50
PS(18:0_20:4)	PS(15:0/18:1(d7))	810.6	283.3	-50
PS(18:0_22:6)	PS(15:0/18:1(d7))	834.6	283.3	-50
PS(18:1/18:1)	PS(15:0/18:1(d7))	786.6	281.3	-50
PS(22:6/22:6)	PS(15:0/18:1(d7))	878.5	327.3	-50
Stearic acid	Arachidonic acid-d8	283.2	283.2	-10

Note: IS - Internal Standard;

The percent of samples with missing area ratio is calculated across the entire set of samples, including the pooled control samples

**Table 3:** GalCer/GlcCer acquisition parameters

Name	Internal Standard	Q1 m/z	Q3 m/z	CE (V)
alpha-GalCer(d18:1/16:0)	GalCer(d18:1/15:0)	700.6	264.3	32
alpha-GalCer(d18:1/18:0)	GalCer(d18:1/15:0)	728.6	264.3	32
alpha-GalCer(d18:1/20:0)	GalCer(d18:1/15:0)	756.6	264.3	32
alpha-GalCer(d18:1/22:0)	GalCer(d18:1/15:0)	784.7	264.3	32
alpha-GalCer(d18:1/22:1)	GalCer(d18:1/15:0)	782.7	264.3	32
alpha-GalCer(d18:1/24:0)	GalCer(d18:1/15:0)	812.7	264.3	32
alpha-GalCer(d18:1/24:1)	GalCer(d18:1/15:0)	810.7	264.3	32
alpha-GalCer(d18:2/18:0)	GalCer(d18:1/15:0)	726.6	262.3	32
alpha-GalCer(d18:2/20:0)	GalCer(d18:1/15:0)	754.6	262.3	32
alpha-GalCer(d18:2/22:0)	GalCer(d18:1/15:0)	782.7	262.3	32
Cholesteryl galactoside	GlcCer(d18:1(d5)/18:0)	566.6	369.3	13
Cholesteryl glucoside	GlcCer(d18:1(d5)/18:0)	566.6	369.3	13
Galactosylsphingosine	Glucosylsphingosine(d5)	462.2	282.3	18
GalCer(d18:1/15:0)	IS	686.3	264.3	32
GalCer(d18:1/16:0)	GalCer(d18:1/15:0)	700.6	264.3	32
GalCer(d18:1/18:0)	GalCer(d18:1/15:0)	728.6	264.3	32
GalCer(d18:1/20:0)	GalCer(d18:1/15:0)	756.6	264.3	32
GalCer(d18:1/22:0)	GalCer(d18:1/15:0)	784.7	264.3	32
GalCer(d18:1/22:1)	GalCer(d18:1/15:0)	782.7	264.3	32
GalCer(d18:1/24:0)	GalCer(d18:1/15:0)	812.7	264.3	32
GalCer(d18:1/24:1)	GalCer(d18:1/15:0)	810.7	264.3	32
GalCer(d18:2/18:0)	GalCer(d18:1/15:0)	726.6	262.3	32
GalCer(d18:2/20:0)	GalCer(d18:1/15:0)	754.6	262.3	32
GalCer(d18:2/22:0)	GalCer(d18:1/15:0)	782.7	262.3	32
GlcCer(d18:1(d5)/18:0)	IS	733.6	269.3	32
GlcCer(d18:1/16:0(d3))	IS	703.6	264.3	32
GlcCer(d18:1/16:0)	GlcCer(d18:1/16:0(d3))	700.6	264.3	32
GlcCer(d18:1/18:0)	GlcCer(d18:1(d5)/18:0)	728.6	264.3	32
GlcCer(d18:1/20:0)	GlcCer(d18:1(d5)/18:0)	756.6	264.3	32
GlcCer(d18:1/22:0)	GlcCer(d18:1(d5)/18:0)	784.7	264.3	32
GlcCer(d18:1/22:1)	GlcCer(d18:1(d5)/18:0)	782.7	264.3	32
GlcCer(d18:1/24:0)	GlcCer(d18:1(d5)/18:0)	812.7	264.3	32
GlcCer(d18:1/24:1)	GlcCer(d18:1(d5)/18:0)	810.7	264.3	32
GlcCer(d18:2/18:0)	GlcCer(d18:1(d5)/18:0)	726.6	262.3	32
GlcCer(d18:2/20:0)	GlcCer(d18:1(d5)/18:0)	754.6	262.3	32
GlcCer(d18:2/22:0)	GlcCer(d18:1(d5)/18:0)	782.7	262.3	32
Glucosylsphingosine	Glucosylsphingosine(d5)	462.2	282.3	18
Glucosylsphingosine(d5)	IS	467.2	287.3	18
Sitosteryl galactoside	GlcCer(d18:1(d5)/18:0)	594.6	397.4	13
Sitosteryl glucoside	GlcCer(d18:1(d5)/18:0)	594.6	397.4	13

Note: IS - Internal standard

### Cell line generation

Cell line engineering of A549 cells to generate homozygous LRRK2 R1441G (CGC/GGC) knock-in, homozygous *LRRK2* KO, and homozygous *GBA*1 knock-out was performed using CRISPR/Cas9. Human LRRK2 G2019S and LRRK2 KO iPSC lines were generated in human iPSCs obtained from a female clone from Thermo Fisher (#A18945). Sequence information for generating targeting gRNA, ssODN donor and PCR primers are as follows:

**Table:** 

**LRRK2 R1441G knock-in (A549)**	**sequences**
sgRNA	AAGAAGAAGCGCGAGCCUGG
Donor Sequence	AAATGTGTGCCAACGAGAATCACAGGGGAAGAAGAAGCGCCAGCCTGGAGGGAAAGACACAAAACCCTCTTGTGTTTGCTTTCAAA
Forward PCR Primer (5’-3’)	AGGCATGAAGATGGGAAAGGA
Reverse PCR Primer (5’-3’)	GGAACCCTCGCTTATTCAGGA
**LRRK2 knock-out (A549)**	**sequences**
sgRNA 1	GGGGACTGTCGACGGTGATCGGT
sgRNA 2	GGTCCTAAACCTGGTCGCAAAGA
Donor Sequence	n/a
Forward PCR Primer (5’-3’)	AGTCCGCTGAGTCAGTTTCTTC
Reverse PCR Primer (5’-3’)	GGGCTCTTCATCCCGTTTACA
**GBA1 knock-out (A549)**	**sequences**
sgRNA	CCAUUGGUCUUGAGCCAAGU
Donor Sequence	n/a
Forward PCR Primer (5’-3’)	GCAAGATTGACAGGCCCAAG
Reverse PCR Primer (5’-3’)	GGCTAATGGCTGAACCGGAT
**LRRK2 G2019S Knock-in (hIPSC)**	**sequences**
sgRNA 1	ATTGCAAAGATTGCTGACTA
sgRNA 2	GTCAGCAATCTTTGCAATGA
Donor Sequence	TGTGCCCTCTGATGTTTTTATCCCCATTCTACAGCAGTACTGAGCAATGCTGTAATCAGCAATCTTTGCAATGATCGCAGCATTGGGATACAGTGTGAAAAGCAGCACATTGTGGGGTTTCAGGTC
Forward PCR Primer (5’-3’)	AGGGACAAAGTGAGCACAGAA
Reverse PCR Primer (5’-3’)	AGGAAAACAGAGTCTATCACATTGT
**LRRK2 Knock-out (hIPSC)**	**sequences**
sgRNA	AGAAACGCTGGTCCAAATCCTGG
Donor Sequence	n/a
Forward PCR Primer (5’-3’)	CATAAACAGGCGGGCGTGG
Reverse PCR Primer (5’-3’)	CTCAGCTGCTTTCTGCCCTAA

Human LRRK2 G2019S and *LRRK2* KO iPSC lines as described in table above were generated by using a nucleofection-based RNP approach to introduce Cas9 (NEB # M0646M) and sgRNAs against LRRK2 (crRNAs ordered from IDT) were introduced into iPSCs via nucleofection (Lonza P3 kit # V4XP-3032) as described before [41]. sgRNAs were designed using the Broad Institute design tool based on a previous studyb [42]. Clones were screened by T7 endonuclease and positive clones were further screened by TOPO cloning (Thermo cat #450030) to identify precise mutations. Clones with indels introduced into both alleles that result in a null mutation were grown up and used as LRRK2 KO clones.

To enable the rapid isolation of lysosomes using immunopurification, A549 cells; wild type, *LRRK2* KO, LRRK2 R1441G and *GBA1* KO cells were transduced with lentivirus carrying the transgene cassette for expression of TMEM192-3x-HA. Stable expression cells were selected using resistance to Hygromycin B (ThermoFisher #10687010) supplied in growth medium at 200 µg/mL for 21 days. Following selection cells were screened for the stable expression of TMEM192-3x-HA (Cell Signaling Technology, 3724 S, 1:100) in lysosomes by quantifying the percentage of cells with co-localization of anti-HA and anti-LAMP2 (Abcam, ab25631, 1:100) by immunofluorescence, and by monitoring cell lysates for expression TMEM192-3x-HA (~ 30 kDa, Cell Signaling Technology, 3724 S, 1:1000) by western blot.

### Human iMicroglia differentiation

Human iPSC-induced microglia were generated as previously described with minor modifications [41]. Briefly, human iPSCs were routinely passaged as clumps onto Matrigel-coated plates with mTeSR + media (StemCell Technologies #85850) according to manufacturer instructions. iPSCs were first differentiated into hematopoietic progenitor cells using a combined strategy of incorporating primitive hematopoiesis via a WNT switch [43]and sorting and replating CD235a + cells in Medium B from a commercially available kit (StemCell Technologies #05310). Briefly: On Day 0 iPSCs were singularized with Accutase treatment for five minutes, pelleted at 300 x g for 5 min, and seeded and cultured according to Guttikonda et al. On Day 3 cells were singularized using Accutase (~ 10 min) and labeled with anti-CD235a-APC conjugated antibody and sorted using a BD FACS Aria. Cells were replated in prepared Medium B from Stemcell technologies. The manufacturer’s protocol was followed for an additional 7–10 days and numerous CD43 + progenitor cells (HPC) are collected and harvested during day 10–14. HPCs positive for the markers CD43 and negative for CD11b were transferred to a plate containing primary human astrocytes and co-cultured using media C adapted from a previous study [44]. Once floating cells in co-culture were > 90% mature microglia, cells were plated in fibronectin (Millipore FC010-10MG) -coated (5 µg/mL) flat-bottom 48-well tissue culture plates (Corning #3548) for 3 days prior to experiments. Full characterization of human iPSC-derived microglia and additional details on the differentiation protocol have been published previously [41]. iPSC-derived microglia were grown “C+++ media” composed of IMDM (GIBCO) media supplemented with 10% defined FBS (GIBCO), 1% Penicillin/Streptomycin (GIBCO), 20 ng/mL of hIL3 (Peprotech), 20 ng/mL of hGM-CSF (Peprotech) and 20 ng/mL of hM-CSF (Peprotech). For experiments, the previously-described C+++ media and “NGD + media” adapted from Muffat et al. were used [45].

### Analysis of lysosomal proteolysis using DQ-BSA-based assay

A549 cells were seeded in 96 well poly-lysine coated plate, and then treated with DNL151 for 3 days. Cells were loaded with culture media containing 10 mg/mL of DQ Red BSA (Invitrogen D12051) and NucBlue (Thermo R37605) for 30 min., and then washed once and replaced with fresh culture media, followed by live cell imaging with 40X confocal (Opera Phenix high content imager). The image analysis was done with 9-hour time point after loading. Image analysis was performed using Harmony software. Spot analysis was used to identified DQ-BSA positive spots within the cell, with “corrected spot intensity” quantified. The Sum of corrected spot intensity per cell was used to measure DQ-BSA signals.

### Analysis of endo-lysosomal GCase activity using FQ6

LysoFQ-GBA was recently described and obtained from the laboratory of David Vocadlo [46]. Briefly, cells were seeded into 96-well PDL-coated plates. FQ6 was added to cells for 1 h under standard incubation conditions (humidified, 37 C, 5% CO2) at a final concentration of 5µM for A549 cells and at 10. µM for iMG using ‘C+++’ media. After 1 h, cells were then rinsed 3x with 37 °C wash buffer (live cell imaging solution (Invitrogen #A14291DJ) supplemented with 5.55 mM Glucose), counterstained with NucBlue (ThermoFisher) for staining nuclei for 10 min. in imaging solution (wash buffer supplemented with 5% fetal bovine serum) and then imaged in the same buffer.

Imaging was performed on a Perkin Elmer Opera Phenix High Content Imaging System with acquisition of FQ6 (ex:488 nm, em:500–550 nm) and NucBlue(DAPI) (ex:375 nm, em:435–480 nm using a 40X water immersion objective. Analysis FQ6 signal “GCase activity” was performed using Harmony software. Harmony Spot Analysis was used to identify FQ6 positive spots within the cell, quantify the “corrected spot intensity”, and then the sum of corrected spot intensities per field of view was normalized to total number of nuclei in the field (to account for differences in cell number) and reported as “SUM Corrected Spot Intensity”.

### Endolysosomal pH measurement

Endolysosomal pH was determined by the fluorescence ratio between a pH-sensitive dye Oregon Green 488 conjugated dextran, 10 kDa (ThermoFisher D7171) and a pH-insensitive dye TMR conjugated dextran, 10 kDa (ThermoFisher D1868). A549 cells were seeded in 96 well poly-lysine coated plate and loaded with 100 µg/mL of Oregon Green 488 Dextran and 100 µg/mL of TMR conjugated dextran overnight, and chased in media without dye for 4 h before imaging. Cells were imaged with 40X confocal (Opera Phenix high content imager) in live cell imaging buffer (ThermoFisher A59688DJ) supplement with 4.5 mg/L glucose, non-essential amino acids solution (Gibco 11140050), MEM amino acids solution (Gibco 11140050) and GlutaMax (Gibco 35050061). pH calibration was performed to generate the pH standard curve. pH standard buffers (in mM: 140 KCl, 1 MgCl_2_, 5 NaCl, 10 sodium acetate, 10 HEPES, and 15 MES) were prepared at varied pH ranging from 4.0 to 6.0 supplemented with 30 µM Nigericin and 15 µM Monensin. Image analysis was performed using Harmony software. Spot analysis was used to identify TMR positive spots within the cell, and the ratio of spot intensity between Oregon green and TMR was used to calculate endolysosomal pH.

### Lysosomal isolations

Lysosomes were isolated from A549 cells with stable expression of TMEM192-3x-HA. Rapid isolation of lysosomes was performed as described previously [47]. Briefly, cells were seeded into 15 cm tissue culture plates at a density of ~ 5 × 10^6 cells/plate. After 72 h under standard incubation (humidified, 37 C, 5% CO2) cells were harvested. Medium was aspirated and cells rinsed 1x with ice-cold PBS. Cells were then scrapped into ice-cold KBPS (136 mM KCl, 10 mM KH_2_PO_4_, pH 7.25) and gently pelleted (300 x g, 5 min., 4 °C). Cell pellets were reconstituted into 500 µM ice-cold KBPS+ (47mL KPBS, 3mL Opti-prep (Sigma D1556), and fresh protease inhibitor cocktail) and sheered by passage through a 21gauge needle. Post nuclear supernatants (PNS) were generated by centrifugation at 800 x g, 10 min., 4 °C. The PNS was transferred to a clean Eppendorf tube and volume adjusted to 1 mL with KBPS. For rapid immunoprecipitation 80µL anti-HA magnetic resin (Thermo-Fisher #88836) is added and samples are rotated end over end at 4 °C for 15 min. Magnetic resin is captured onto manual MACS magnetic separator (Miltinyi) and the captured lysosomes are washed 2x with 1 mL KPBS+. Lysosomes are then processed immediately for downstream analysis.

### MSD (Meso scale Discovery) assay to measure LRRK2, pS935 LRRK2 and pT73 Rab10 levels

LRRK2, pS935 LRRK2 and pT73-Rab10 MSD assays were previously established [21]. Capture antibodies were biotinylated using EZ-Link™ NHS-LC-LC-Biotin (Thermo Fisher, #21343), and detection antibodies were conjugated using Sulfo-TAG NHS-Ester (MSD, R31AA-1). 96-well MSD GOLD Small Spot Streptavidin plates (MSD L45SSA-1) were coated with 25 µL of capture antibody diluted in Diluent 100 (MSD, R50AA-2) for 1 h at room temperature with 700 r.p.m shaking. After three washes with TBST, 25 µL samples were added each well and incubated at 4 °C overnight with agitation at 700 r.p.m. After three additional washes with TBS-T, 25 µL of detection antibodies were added to each well diluted in TBS-T containing 25% MSD blocker A (MSD R93AA-1) together with rabbit (Rockland Antibodies D610-1000) and mouse gamma globin fraction (D609-0100). After 1 h incubation at room temperature at 700 r.p.m. and three washes with TBS-T, 150 µL MSD read buffer (MSD R92TC, 1:1 diluted with water) was added, and plates were read on the MSD Sector S 600.

**Table:** 

Assay	Antibody type	targets	Vendor	Cat No.	Concentration (ug/mL)
pS935 LRRK2	Capture	pS935 LRRK2	Abcam	ab133450	0.5
	Detection	Total LRRK2	BioLegend	808,201	1
Total LRRK2	Capture	Total LRRK2	BioLegend	844,401	0.5
	Detection	Total LRRK2	BioLegend	808,201	1
pT73 Rab10	Capture	pT73 Rab10	Denali	19 − 4	1
	Detection	Total Rab10	Abcam	ab181367	2

### siRNA-mediated KD of Rab GTPases

A549 cells were transfected with Dharmacon SMARTpool siRNA targeting 14 Rab GTPases, LRRK2, GBA1 and non-targeting scramble control (Horizon Discovery, Cambridge, United Kingdom), using DharmaFECT 1 (Horizon, T-2001-01). Cells were collected 3 days after transfection for protein or mRNA analysis.

**Table:** 

Targets	Catalog Number	Sequence
ON-TARGETplus Non-targeting Control	D-001810-10	UGGUUUACAUGUCGACUAA
RAB3A	L-009668-00	GAAGAUGUCCGAGUCGUUG
RAB3B	L-008825-00	GGACACAGACCCGUCGAUG
RAB3C	L-008520-00	UGAGCGAGGUCAACAUUUA
RAB3D	L-010822-00	GUUCAAACUGCUACUGAUA
RAB5A	L-004009-00	GCAAGCAAGUCCUAACAUU
RAB5B	L-004010-00	GGAGCGAUAUCACAGCUUA
RAB5C	L-004011-00	UCAUUGCACUCGCGGGUAA
RAB8A	L-003905-00	CAGGAACGGUUUCGGACGA
RAB8B	L-008744-00	GCAAUUGACUAUGGGAUUA
RAB10	L-010823-00	GCAAGGGAGCAUGGUAUUA
RAB12	L-023375-02	CAUUUGAUGAUUUGCCGAA
RAB29	L-010556-00	GAGAACGGUUUCACAGGUU
RAB35	L-009781-00	GAUGAUGUGUGCCGAAUAU
RAB43	L-028161-01	GGAUGAGAGGGCACCGCAA
LRRK2	L-006323-00	GAAAUUAUCAUCCGACUAU
GBA	L-006366-00	CCAAUUGGGUGCGUAACUU

### RT-PCR-based analysis of Rab expression

The total RNA was extracted from cells using RNeasy Plus Micro Kit (QIAGEN, Hilden,

Germany, #74034). cDNA was synthesized from 1 ~ 2 µg of RNA using Superscript IV VILO master mix (Thermo Fisher #11756050). The cDNA was diluted 3-fold and 1 µL of diluted cDNA was used as template. To measure the relative expression levels of mRNAs by RT-qPCR, Taqman Fast Advanced Master Mix (Thermo Fisher #4444557) was used, together with genespecific primers using TaqMan Assays (Thermo Fisher). GAPDH was used as the housekeeping gene. The PCR reaction was run using QuantStudio™ 6 Flex Real-Time PCR System, 384-well (Thermo Fisher). Gene expression was analyzed using 2^^(delta−delta Ct)^ method with GAPDH as internal controls.

**Table:** 

Taqman assay ID	Gene name	Dye
Hs00923221_m1	RAB3A	FAM-MGB
Hs01001137_m1	RAB3B	FAM-MGB
Hs00384846_m1	RAB3C	FAM-MGB
Hs00758197_m1	RAB3D	FAM-MGB
Hs00702360_s1	RAB5A	FAM-MGB
Hs05027271_g1	RAB5B	FAM-MGB
Hs00904926_g1	RAB5C	FAM-MGB
Hs00180479_m1	RAB8A	FAM-MGB
Hs00213006_m1	RAB8B	FAM-MGB
Hs00794658_m1	RAB10	FAM-MGB
Hs01391604_m1	RAB12	FAM-MGB
Hs01026316_m1	RAB29	FAM-MGB
Hs00199284_m1	RAB35	FAM-MGB
Hs03006628_gH	RAB43	FAM-MGB
Hs01115057_m1	LRRK2	FAM-MGB
Hs00986836_g1	GBA	FAM-MGB
Hs99999905_m1	GAPDH	VIC

### Collection and analysis of exosomes from human urine from DNL201 Ph1b study

Urine samples were collected prior to dosing and on day 28 following administration of placebo or DNL201 (50 mg TID) to human subjects with PD from our Ph1b study as previously described (NCT03710707) [14]. We have complied with all relevant ethical regulations, and the study protocol, amendments, and informed consent form were reviewed and approved by the institutional review board/independent ethics committee at each CRU: Quorum Review, Inc, Henry Ford Health System Institutional Review Board, University of Pennsylvania Institutional Review Board, and Western Institutional Review Board. Informed consent was obtained from all participants. Within 30 min of the end of the collection period, samples were centrifuged at 2500xg for 15 min at 4 °C. Urine was transferred to aliquot tubes and stored at − 80 °C. Samples were thawed on ice and centrifuged at 1000 x g for 10 min at 4 °C to remove particulates. 500 µL urine was transferred for exosome isolation according to the EVTRAP method previously described [40]. Following elution, samples were processed for LC-MS/MS analysis: single step extraction of pelleted exosomes was performed by adding 400 µL of LC-MS/MS grade methanol containing 2 µL internal standard mix solution. Resultant supernatants were transferred into 96 well plates with glass inserts and dried under a constant stream of N_2_. Dried samples were stored in -80 °C until analysis, and analyzed as described above via LC-MS/MS.

### Western blot analysis

Cell lysates were prepared by incubating with NuPage LDS Sample Buffer (ThermoFisher, NP0007) and NuPAGE™ Sample Reducing Agent (ThermoFisher, NP0004) for 5 min at 95 °C to denature samples. Lysates were loaded onto NuPAGE 4–12% Bis-Tris gels (Invitrogen). Proteins were transferred to nitrocellulose membranes for 7 min. (Trans-Blot Turbo Transfer System, Bio-Rad). Membranes were blocked with Rockland blocking buffer, incubated with primary antibody overnight at 4 °C, and then with secondary antibodies (1:20,000, LI-COR) for 1 h at room temperature. LI-COR Odyssey system was used for western blot detection and quantitation. The primary antibodies used were anti-HA (1:500, CST #3724), anti-GCase (1:500, Sigma G4046, anti-LAMP1 (1:500, CST #9091)), anti-actin (1:5000, Sigma, A2228). The secondary antibodies used were Donkey anti-mouse 680RD (1:20000, LI-COR #926-68072), Donkey anti-rabbit 800CW (1:20000, LI-COR #926-32213).

### Analysis of association of LRRK2 variants with urine BMP levels in human subjects

A total of 1,069 samples were available for statistical testing of the association of *LRRK2* variants on urine BMP levels (themselves available as ng/mg creatinine). Four BMP phenotypes were tested: the directly measured levels of total 22:6 and total 18:1 species, and derived ratios of 2,2’ 22:6/total 22:6, and total 22:6/total 18:1. To perform association testing, natural log-transformed BMP phenotypes were fit in a linear model consisting of age, sex, disease status, and the first 5 principal components derived from the WGS data (“Model 1”, see below). The residuals from this model were then inverse normal transformed using the blom() function from the “rcompanion” R package and used in association testing against the *LRRK2* G2019S (rs34637584) and N551K (rs7308720) variants. Association testing was carried out using an additive model in plink v1.9 [48]. Finally, sensitivity analyses were conducted to examine the impact of removing the adjustment by disease status (“Model 2”), or adding adjustment by *GBA* N370S/N409S status (rs76763715; “Model 3”), *LRRK2* N551K status (for G2019S inference only; “Model 4”) or LRRK2 G2019S status (for N551K inference only, “Model 5”). The choice to examine the effect of adjusting by *LRRK2* G2019S and *GBA* N370S/N409S was informed by the large amount of carriers of these major PD risk-conferring variants, as they comprised 27% and 22% of the 1,069 samples, respectively.

### Analysis of CSF lipid levels from human subjects

The lipid analysis on human CSF reported in this manuscript was generated as part of a larger metabolomic characterization whose methods have been previously reported [49]. Raw data from this study are available via online LCC data request at https://www.michaeljfox.org/news/lrrk2-cohort-consortium.

### CSF sample acquisition and human participants

CSF samples used in the analyses presented in this article were obtained from the MJFF-sponsored LRRK2 Cohort Consortium (LCC). For up-to-date information on the study, visit https://www.michaeljfox.org/biospecimens. The LRRK2 Cohort Consortium is coordinated and funded by The Michael J. Fox Foundation for Parkinson’s Research. Previous publications have described the LRRK2 Cohort Consortium in detail [50]. CSF was collected as part of the LRRK2 cross-sectional study according to guidelines provided in the biologics manual which can be found at http://mjff.prod.acquia-sites.com/sites/default/files/media/document/LRRK2%20Cohort%20Consortium%20Biologics%20Manual%20Final%201.1.pdf.

### Data analysis for LC-MS based measurements

Peak areas for analytes detected were first normalized with spiked in internal standard areas. Pairing of specific analytes to surrogate internal standards are used are listed in supplementary Tables 1–3.

### Statistical analysis of lipid levels from tissues and fluids of LRRK2 KO/G2019S mice

Lipid levels were log-transformed and analyzed using analysis of covariance (ANCOVA) models with terms for genotype (and terms for treatment group, or genotype x treatment interaction if applicable), and adjustment for key covariates (sex, age and/or weight). Specific covariates adjustments were study dependent. Effect sizes between groups were estimated via geometric mean ratios, and corresponding 95% confidence intervals (CI), and statistical significance was assessed at two-sided 5% significance levels. For each sample type, adjusted p-values corrected for multiple comparisons were derived using the Benjamini-Hochberg (BH) method.

### Analysis of human CSF from the LCC cohort

Raw area ratios of CSF lipids detected were normalized to equalize the median absolute deviation to 1 and log2 transformed using the limma package (version 3.46). Analysis of % differences in lipid abundance by LRRK2 and disease status was performed by robust linear model procedure using Age and Sex and covariates and the following three-way interaction model: log2(area ratio) ~ LRRK2*PD status*Sex + Sex*Age. Post-hoc pairwise comparisons were analyzed using the estimated marginal means function provided in the emmeans r package (version 1.7.5). Conversion to % changes were performed using the update function provided in the stats r package (version 4.0.2). Correlation analysis between BMP(22:6/22:6) and GlcCer(d18:1/24:1) species stratified by LRRK2 and PD disease status was performed using the stat_cor function provided by ggpubr package (version 0.4.0). Visualization of heatmaps and boxplots were performed using the ggplot2 (version 3.3.6) and the ComplexHeatmap (version 2.6.2) packages. Data analyses above were performed using the R statistical software (version 4.0.2; R Core Team 2020).

## Results

### LRRK2 kinase activity regulates urinary BMP levels by modulating the release of BMP- and glycosphingolipid-containing vesicles from kidney

While BMP has been identified as a potential biomarker to assess LRRK2-dependent effects on lysosomal homeostasis in the clinic, a lack of understanding around what changes in urine BMP levels reflect about lysosomal function in tissue has limited its utility. To better understand the mechanism by which LRRK2 activity regulates urinary BMP levels, we explored the impact of *LRRK2* deletion on BMP in mouse urine and kidney, a likely key tissue source of urine BMP. Consistent with previous reports, we confirmed that BMP(22:6/22:6) levels were significantly reduced in urine from *LRRK2* KO mice compared to wildtype (WT) controls (Fig. 1A) [31]. Mechanistically, LRRK2 could regulate BMP synthesis in the kidney, and loss of LRRK2 would therefore result in a similar reduction of BMP in tissue. Alternatively, LRRK2 may drive the secretion of BMP-containing intraluminal vesicles through exosome release to the extracellular space, as shown in other lysosomal dysfunction models [51], and loss of LRRK2 would instead be expected to result in the accumulation of BMP in kidney. To discriminate between these possibilities, we performed imaging mass spectrometry (IMS) to assess the spatial distribution of BMP in kidneys from WT and *LRRK2* KO mice, which revealed accumulation of BMP(22:6/22:6) within the renal cortex and outer medulla of *LRRK2* KO mice compared to WT controls (Fig. 1B). We confirmed that BMP(22:6/22:6) levels were significantly increased in *LRRK2* KO mouse kidney by quantifying BMP levels from dissected renal cortex and medulla using LC-MS/MS, with higher levels of BMP observed in the renal medulla consistent with our findings using IMS (Supplementary Figure S1A).

These data suggest that LRRK2 may regulate exosome secretion more broadly and therefore impact levels of additional lipids enriched in ILVs beyond BMP, including BMP-related lipid and GSL species [31]. To test this, we performed targeted lipidomics on urine and kidney from WT and *LRRK2* KO mice using LC-MS/MS. Similar to the reduction in BMP observed in urine from *LRRK2* KO mice, levels of GlcCer were also decreased (Fig. 1C). In contrast, we observed a significant accumulation of multiple species of GSLs and components of the BMP pathway, including hemi-BMP, lysophosphatidylglycerol (LPG) and phosphatidylglycerol (PG), in both kidney regions examined from *LRRK2* KO mice (Fig. 1D and Supplementary Table S1). Based on these data, we hypothesized that pathogenic variants that increase LRRK2 kinase activity, such as the LRRK2 G2019S variant, would reduce BMP and GSL levels in kidney and that LRRK2 kinase inhibition would, conversely, increase levels of these lipid species in kidney. To assess this, we dosed LRRK2 G2019S KI and WT littermates with either vehicle or a tool LRRK2 kinase inhibitor (MLi-2) in diet for 35 days and assessed the consequences on lipid levels in kidney and urine using LC-MS/MS [51, 52]. Both pharmacokinetics (unbound concentration of the MLi-2) and LRRK2 pS935 analysis demonstrated a high level (> 90%) of LRRK2 kinase inhibition (Supplementary Fig. S1B and C). In kidney, we observed a significant reduction in BMP and GSL levels in LRRK2 G2019S mice compared to WT littermates (Fig. 1E and Supplementary Fig. S1D), and LRRK2 inhibition increased levels of BMP-related lipids and several classes of GSL (Fig. 1E and Supplementary Figure S1D, and Supplementary Table S1). The levels of urine BMP were significantly reduced compared to vehicle treated mice following LRRK2 kinase inhibition in both LRRK2 G2019S KI and WT mice (Fig. 1F, Supplementary Fig. S1E, Supplementary Table S1), consistent with previous findings in both preclinical and clinical studies [14, 31, 53]. Beyond changes in BMP, several species of GlcCer were also reduced following LRRK2 kinase inhibition in mouse urine (Fig. 1F, Supplementary Table S1).

Carriers of the kinase-activating LRRK2 G2019S variant have been reported to have elevated urine BMP levels, and LRRK2 kinase inhibition resulted in a dose-dependent reduction in urine BMP levels in healthy volunteers and PD patients [14, 32]. To further investigate the relationship between disease risk, LRRK2 kinase activity, and BMP levels in humans, we used publicly available urine BMP data and whole-genome sequencing (WGS) data in the Parkinson’s Progression Markers Initiative (PPMI). After restricting WGS PPMI samples to those with available urine BMP data and those of predicted European ancestry (see Materials and Methods), 1,069 samples (derived from subjects with and without PD) were available for data analysis. This dataset was enriched for carriers of the PD risk variants LRRK2 G2019S (*N* = 289; 27%) and GBA N370S/N409S (*N* = 238; 22%). In models adjusting for sex, age, disease status and principal components derived from the WGS data, urine levels of BMP(22:6/22:6) were significantly increased in carriers of the PD risk variant G2019S (*p* = 5.78E-82; Fig. 1G and Supplementary Table S2), consistent with previous studies [32, 54]. Our recent work showed that the LRRK2 protective haplotype N551K-R1398H is associated with reduced LRRK2 kinase activity, as reflected by the levels of phosphorylated Rab10 (pT73 Rab10) in both cellular models and human subjects [21]. We next assessed whether the LRRK2 N551K protective variant impacted BMP levels in urine and observed a significant decrease in urine BMP(22:6/22:6) levels in carriers of the PD protective variant N551K (*p* = 7.33E-4; Fig. 1G and Supplementary Table S2). These relationships were maintained (consistent direction of effect and *p* < 0.05) in sensitivity analyses that removed the adjustment by disease status or additionally adjusted for GBA N370S/N409S status, N551K status (for the G2019S effect) or G2019S status (for the N551K effect) (Supplementary Table S2). This analysis provides evidence that genetic variants that either increase or decrease LRRK2 kinase activity have the concurrent effect on extracellular levels of BMP in urine. Additionally, the reduction in urine BMP observed in carriers of N551K LRRK2 protective allele further supports therapeutic strategies focused on reduction of LRRK2 kinase inhibition.

Our collective results suggested that LRRK2-dependent secretion of BMP-containing vesicles into urine is driven by LRRK2 kinase activity, and we next directly assessed this in human subjects dosed with a LRRK2 inhibitor from our clinical study. Using urine samples obtained from human subjects dosed with the LRRK2 kinase inhibitor DNL201 in our recent phase 1b clinical study, we isolated extracellular vesicles (EVs) and analyzed their lipid content via mass-spectrometry [14]. BMP was readily detected from EVs isolated from human urine, consistent with previous findings that BMP is present in exosomes [55]. We observed a trend toward reduction in total lipid levels and in BMP specifically in EVs isolated from urine from subjects treated with DNL201 (*n* = 11) compared to the placebo group (*n* = 7; Fig. 1H), although these results did not reach statistical significance. While the sample size was limited and not specifically powered for this assessment, these data are consistent with our hypothesis that LRRK2-dependent regulation of exosome release may be a key mechanism by which LRRK2 inhibition reduces BMP levels in urine.

### LRRK2 kinase activity regulates glycosphingolipid and BMP levels in a cell type-specific manner in mouse brain

Having established that LRRK2 regulates the intracellular levels of BMP and GSLs in kidney, we next wanted to assess whether LRRK2 similarly regulates BMP levels in the CNS. We first assessed the levels of BMP(22:6/22:6) throughout the brain of *LRRK2* KO mice and WT littermates using imaging mass spectrometry. Overall, we did not observe gross differences in brain BMP levels between WT and *LRRK2* KO mice, in contrast to the effects observed in the kidney (Fig. 2A and Supplementary Fig. S2A-C). While this global analysis suggests that LRRK2 does not profoundly impact BMP levels in the brain, we reasoned that LRRK2 may impact BMP concentrations in a cell-type specific manner given the low expression of LRRK2 throughout the CNS. Previous analyses suggest that LRRK2 expression is highest in mouse astrocytes, with moderate expression in mouse microglia and lower expression in cortical neurons [21, 27]. We employed a fluorescence-activated cell sorting (FACS)-based method to isolate enriched populations of astrocytes, microglia, and neurons from *LRRK2* KO and LRRK2 G2019S KI mice and assessed lipid changes using mass-spectrometry [56]. We observed the most profound lipid changes in astrocytes from *LRRK2* KO mice compared to other CNS cell types, with reductions in several classes of GSLs (Fig. 2B and C, Supplementary Fig S2D and E, and Supplementary Table S2). Consistent with our whole brain analysis, BMP levels were not significantly altered in astrocytes, neurons or microglia isolated from *LRRK2* KO mice (Fig. 2D). Together, these data indicate that LRRK2 deletion influences GSL levels in the brain and, in contrast to effects observed in kidney, does not impact BMP levels.

To assess whether LRRK2 hyperactivity affected BMP and GSL levels in brain, we performed a similar targeted lipidomic analysis from FACS-enriched CNS cells from LRRK2 G2019S KI mice and WT littermate controls and measured lipid levels in CNS cells isolated from young (5-6-month-old) and aged (18-month-old) mice. Many GSLs accumulated in astrocytes and microglia from LRRK2 G2019S mice in both age groups assessed (Fig. 2E and H and Supplementary Table S3), including several species of GlcCer and GalCer (Fig. 2F and I). BMP levels, however, were only significantly altered in aged LRRK2 G2019S astrocytes, showing a modest reduction compared to astrocytes from WT mice (Fig. 2G). GSL dysregulation was most apparent in microglia isolated from LRRK2 G2019S mice (Fig. 2H), consistent with emerging data from LSDs that suggest microglia are particularly susceptible to defects in lysosomal function [56, 57]. The changes in GSLs observed in neurons were comparatively modest (Supplementary Fig. S2F), which may be attributable to the relatively low expression of LRRK2 reported in mouse neurons compared to glial cells [58]. Our data show that LRRK2 hyperactivity leads to the accumulation of many GSL species, including GlcCer and GalCer, in glial cells and more modestly impacts BMP levels in an age-dependent manner. Together, our results suggest that LRRK2 has a more pronounced effect on GSL levels than BMP in the mouse brain and highlight the potential utility of GSLs as readouts of LRRK2-dependent effects on lysosomal function in the CNS.

### LRRK2 kinase activity regulates lysosomal function and the levels of BMP and GCase substrates in cellular models

Our lipid profiling of LRRK2 G2019S and KO mouse brain highlighted the impact of LRRK2 activity on GSL levels across CNS cell types. To better understand how LRRK2 kinase activity modulates GSLs and to define whether LRRK2-dependent effects on BMP play a role in this regulation, we developed a cell model that endogenously expresses the pathogenic LRRK2 variant R1441G using CRISPR-Cas9. A549 cells were selected based on the model’s high endogenous expression of both LRRK2 and the LRRK2 substrate Rab10 [23], and we focused on the pathogenic LRRK2 R1441G variant given its strong impact on Rab phosphorylation [59]. Using LC-MS/MS, we found that the levels of several species in the BMP pathway, including BMP(22:6/22:6), were significantly elevated in LRRK2 R1441G KI cells compared to WT cells (Fig. 3A and Supplementary Fig. S3A). Further, GSL levels were broadly elevated in LRRK2 R1441G cells compared to WT cells, including nearly all species of GlcCer examined (Fig. 3B). We treated LRRK2 R1441G cells with DNL151, a selective LRRK2 kinase inhibitor currently being assessed in late-stage clinical studies for the treatment of PD (Supplementary Fig. S3B), for 72 h and demonstrated DNL151 treatment fully normalized the levels of GlcCer(d18:1/24:1), the most significantly elevated GlcCer species observed in LRRK2 R1441G cells compared to WT cells (Fig. 3C). Dose-response analysis showed that approximately 80% LRRK2 kinase inhibition, as assessed by the levels of phosphorylated Rab10, was required to reduce GlcCer(d18:1/24:1) levels to that observed in WT cells (Fig. 3D and E). These data are consistent with recent clinical findings with a related LRRK2 inhibitor (DNL201) (Supplementary Fig. S3B), which show that > 80% LRRK2 kinase inhibition is needed to reduce BMP levels in human urine and suggest that this level of inhibition may be necessary to correct lysosomal dysfunction mediated by LRRK2 hyperactivity [14].

To understand the functional impact of BMP and GSL accumulation, we next examined the effects of the pathogenic LRRK2 R1441G variant on endolysosomal protein turnover and pH. Lysosomal proteolysis, assessed using a de-quenched bovine serum albumin (DQ-BSA)-based assay, was reduced by approximately 30–40% in LRRK2 R1441G KI cells compared to WT cells (Fig. 3F and G). Treatment with the LRRK2 kinase inhibitor DNL151 (2 µM) for 72 h fully rescued lysosomal proteolysis, normalizing signal to that observed in WT cells (Fig. 3F and H). The LRRK2 R1441G variant also led to a significant reduction in endolysosomal pH and cathepsin activity as assessed using a pan-cathepsin activity-based probe (Supplementary Fig. S3C and D), consistent with previous work suggesting LRRK2 pathogenic variants can lead to hyperacidification of the endolysosomal compartment [27]. Assessment of endolysosomal cathepsin B and D activity using additional live-cell probes did not show any effect of the LRRK2 R1441G variant on cathepsin B or D activity, suggesting the activity of a different cathepsin may be impacted by LRRK2 hyperactivity (Supplementary Fig. S3E and F). Together, our data demonstrate that mutant LRRK2 perturbs lysosomal proteolysis and triggers lipid storage in cells and establish that LRRK2 kinase inhibition can correct lipid accumulation that correlates with broad improvements in lysosomal function.

### Endolysosomal GCase activity, GlcCer and BMP levels are controlled by LRRK2 kinase activity

To determine whether LRRK2 activity specifically impacts lysosomal lipid homeostasis, we employed an established lysosome immunoprecipitation (Lyso-IP) method that enables rapid isolation of intact lysosomes [47, 60]. Similar to our observations from whole cell extracts, lysosomes isolated from LRRK2 R1441G cells showed broad elevations in GlcCer species compared to lysosomes from WT cells (Fig. 4A and B, Supplementary Fig. S4A and B). DNL151 treatment reduced levels of BMP and all GlcCer species examined, including GlcCer(d18:1/24:1), in lysosomes isolated from LRRK2 R1441G cells to WT levels, demonstrating that LRRK2 kinase inhibition can rescue lysosomal lipid accumulation (Fig. 4B and C and Supplementary Fig. S4A-C).

As elevated LRRK2 activity increased the lysosomal levels of the GCase substrate GlcCer, we hypothesized that the pathogenic LRRK2 R1441G variant impaired GCase activity in these cells. Several studies have assessed the impact of LRRK2 variants on GCase activity and reported conflicting results [61–63]. A clear understanding of the relationship between LRRK2 and lysosomal GCase activity has been hindered by limitations in existing methods to measure GCase activity with respect to sensitivity, selectivity, and spatial resolution. We employed a recently described activity-based probe to measure lysosomal GCase activity (LysoFQ-GBA) that has superior signal-to-noise and lysosomal retention compared to other probes tested to address this question [46]. We confirmed that LysoFQ-GBA specifically detected GCase signal as no detectable signal was observed in *GBA1* KO A549 cells (Fig. 4D). Lysosomal GCase activity was reduced by 40–50% in LRRK2 R1441G cells compared to WT cells, supporting our hypothesis that LRRK2 regulates GlcCer levels by regulating GCase activity (Fig. 4D). LRRK2 R1441G cells accumulated similar GSL species as those observed in *GBA1* KO A549 cells (Fig. 4E), though to a lesser extent, further suggesting that lysosomal GSL accumulation in LRRK2 R1441G cells may result from impaired GCase activity. To determine whether LRRK2 regulated GCase activity by controlling its delivery to lysosomes, we assessed the levels of GCase in isolated lysosomes by western blot analysis. GCase protein levels were not changed in lysosomes isolated from LRRK2 R1441G cells and were significantly elevated in lysosomes isolated from *LRRK2* KO A549 cells (Supplementary Fig. S4D). These data show that the LRRK2 R1441G variant does not impact the delivery of GCase to lysosomes and suggest that LRRK2 hyperactivity may impair GCase activity through alternative mechanisms.

### LRRK2 regulates GCase activity and glycosphingolipid levels in human iPSC-derived microglia

To assess whether LRRK2-dependent regulation of GCase activity and BMP occurs in a disease-relevant CNS cell type, we explored the impact of LRRK2 in human iPSC-derived microglia (iMicroglia) based on their high expression of LRRK2 and recent data showing that microglia-specific regulatory elements may drive the effects of non-coding LRRK2 risk variants in humans [64, 65]. To expand upon our studies in A549 cells, we examined the consequences of a more common LRRK2 pathogenic variant (LRRK2 G2019S) in iMicroglia. We showed that LRRK2 G2019S KI iMicroglia had an approximately two-fold increase in LRRK2 kinase activity, as assessed by levels of phosphorylated Rab10 per molecule of LRRK2 (Fig. 5A and Supplementary Fig. S5A-C). Significant accumulation of the GCase substrate glucosylsphingosine (GlcSph) was observed in LRRK2 G2019S iMicroglia, while GlcSph levels were reduced in *LRRK2* KO iMicroglia compared to WT cells (Fig. 5B). Unlike in our A549 cell studies, we did not observe alterations in GlcCer(d18:1/24:1) with expression of a pathogenic LRRK2 variant in iMicroglia (Supplementary Fig. S5D) which may reflect that these cells have greater levels of acid ceramidase (ASAH1) activity, an enzyme that converts GlcCer to GlcSph. The LRRK2 G2019S variant increased levels of BMP(20:4/20:4) (Fig. 5C). We examined endolysosomal GCase activity using the LysoFQ-GBA substrate and confirmed its specificity in iMicroglia by demonstrating loss of signal following treatment with the GCase inhibitor conduritol β-epoxide (CBE; Fig. 5D). Similar to effects seen in A549 cells, we observed an approximately 50% reduction in endolysosomal GCase activity in LRRK2 G2019S iMicroglia and a trend toward an increase in GCase activity in *LRRK2* KO iMicroglia (Fig. 5D). Together, our data show that LRRK2 regulates GCase activity, substrate catabolism and BMP levels in human iMicroglia, demonstrating that LRRK2-dependent regulation of lysosomal function occurs in a key CNS cell type relevant for PD. The consequences of LRRK2 hyperactivity or deletion on GCase activity or lysosomal homeostasis were not evaluated in other iPSC-derived CNS cell types, including neurons, and additional studies are needed to determine whether comparable alterations are observed across CNS cell types.

### LRRK2 regulates BMP accumulation secondary to its effects on endolysosomal GCase activity

To better define the mechanisms by which LRRK2 hyperactivity can trigger BMP and GCase substrate accumulation in lysosomes, we first set out to better understand what role GCase played in mediating LRRK2-dependent effects on lysosomal lipid homeostasis. To assess whether GCase activity is required for the LRRK2-mediated reduction of GlcCer levels, *GBA1* KO cells were treated with DNL151 (2 µM) for 72 h, and GlcCer levels measured by LC-MS/MS. *GBA1* KO cells showed significant elevations in GlcCer levels compared to WT cells, and LRRK2 inhibition did not significantly impact GlcCer accumulation (Fig. 6A). These data suggest that GCase is essential for LRRK2-dependent lysosomal GlcCer regulation. To test whether restoration of GCase activity was sufficient to rescue LRRK2-mediated GlcCer accumulation, we treated LRRK2 R1441G cells with recombinant GCase (imiglucerase) and evaluated its effects on lipids in isolated lysosomes using LC-MS/MS. Imiglucerase treatment attenuated GlcCer accumulation in isolated lysosomes from LRRK2 R1441G cells (Fig. 6B and Supplementary Fig. S6A). Interestingly, imiglucerase treatment also significantly reduced BMP(20:4/20:4) levels in lysosomes isolated from one clone of LRRK2 R1441G cells (Fig. 6C and Supplementary Fig. S6B), suggesting that BMP accumulation is a secondary consequence of reduced GCase activity. Treatment with recombinant GCase also fully rescued GCase substrate and reduced BMP accumulation in LRRK2 G2019S iMicroglia, demonstrating that LRRK2 acts upstream of GCase to regulate BMP through a similar mechanism in this CNS cell type (Fig. 6D and E). Together, our results show that LRRK2 regulates lysosomal BMP as a downstream response to its effect on GCase activity and reveal a novel mechanism by which LRRK2 regulates lysosomal BMP.

To determine whether GCase deficiency drove broader lysosomal defects observed with LRRK2 hyperactivity, we treated LRRK2 R1441G A549 cells with imiglucerase and assessed its impact on lysosomal proteolysis using the DQ-BSA-based assay. Although LRRK2 kinase inhibition restored lysosomal protein turnover in LRRK2 R1441G KI cells back to levels observed in WT cells, imiglucerase treatment failed to rescue lysosomal proteolysis in these cells (Fig. 6F). These data suggest that LRRK2 hyperactivity drives the accumulation of GCase substrates and BMP through its effects on GCase activity and impairs lysosomal protein turnover through additional mechanisms beyond its effects on GCase.

We next wanted to better define how increased LRRK2 activity led to reduced endolysosomal GCase activity. As LRRK2 phosphorylates a subset of Rab GTPases known to regulate various aspects of membrane trafficking, we explored whether LRRK2 regulates GCase activity through effects on its Rab substrates. We performed a targeted siRNA screen on LRRK2 Rab substrate genes in A549 cells and assessed the impact of RAB knockdown on endolysosomal GCase activity using the LysoFQ-GBA probe. We confirmed that knockdown of GBA1 significantly reduced LysoFQ-GBA signal in these cells as expected and validated previous data suggesting that Rab10 can regulate GCase activity (Fig. 6G and Supplementary Fig. S6C) [63]. RAB3D and RAB12 were identified as additional LRRK2 substrates whose knockdown significantly reduced endolysosomal GCase activity (Fig. 6G). We also assessed the impact of additional Rabs that have not been shown to be direct LRRK2 substrates but have been recently implicated in PD risk, Rab21 and Rab32 [66–68], and found that knockdown of both of these Rabs also significantly impaired endolysosomal GCase activity (Supplementary Fig. S6D and E). We focused our subsequent analysis on the role of the LRRK2 substrates Rab10 and Rab12 in regulating GCase activity and confirmed that deletion of *RAB10* or *RAB12* led to a significant reduction in endolysosomal GCase activity similar to that observed in LRRK2 R1441G cells (Fig. 6H). Significant accumulation of GlcCer(d18:1/24:0) was observed in *RAB12* KO cells compared to WT cells, with *RAB10* KO cells showing a trend toward increased levels of GlcCer (Fig. 6I). Deletion of *RAB10* or *RAB12* also led to accumulation of BMP(22:6/22:6) (Fig. 6I). These data suggest that LRRK2 may regulate GCase activity and secondary lipid accumulation through its substrates Rab10 and Rab12.

### Defects in glucosylceramide catabolism correlate with BMP alterations in CSF from PD patients

While our data demonstrated that pathogenic LRRK2 variants can lead to GSL and BMP accumulation in cellular models, including iMicroglia, we had observed minimal LRRK2-dependent effects on BMP in our analysis of mouse brain. To gain insight into the potential relevance of these LRRK2-dependent lipid changes in the CNS of PD subjects, we measured the levels of various GSLs and BMP in CSF from healthy control and PD subjects with or without *LRRK2* variants using samples collected from the LRRK2 Cohort Consortium (Supplementary Table 4) [49]. BMP and GSL levels were comparable in healthy subjects that carry a *LRRK2* variant compared to non-variant carrier controls (Supplementary Fig. S7A). Significant lipid alterations were observed in CSF from LRRK2-PD patients compared to sporadic PD patients. In contrast to effects observed in urine, BMP(22:6/22:6) levels were reduced by 36% (unadjusted *p* = 0.01) in CSF from PD patients that carry a *LRRK2* variant relative to the PD patients without *LRRK2* variants (Fig. 7A and B). Within the GSL lipid class, GlcCer(d18:1/24:1) increased by 15% (unadjusted *p* = 0.07), and lactosylceramide (LacCer) (d18:1/24:1) decreased by 53%, (unadjusted *p* = 0.07) in *LRRK2* variant PD subjects compared to PD patients that do not carry a variant in *LRRK2* (Fig. 7A and B, Supplementary Fig. S7B). Significant alterations in BMP and GSLs were observed specifically in CSF from *LRRK2* variant carriers with PD, suggesting elevated LRRK2 kinase activity drives BMP and GSL alterations in disease.

Analysis of the substrate levels compared to the levels of product generated has been employed to approximate enzymatic activity and improve sensitivity for identifying genetic associations with lipid phenotypes [69]. Therefore, we quantified the ratio of the sum of GlcCer species to the sum of corresponding ceramide (Cer) species across healthy subjects and PD patients with or without *LRRK2* variants. We observed a significant increase in the total GlcCer/total Cer ratio from 1.9 ± 0.5 (mean ± SD) in PD subjects without LRRK2 variants to 2.2 ± 0.6 (mean ± SD) in PD subjects with a *LRRK2* variant (unadjusted *p* = 0.02) (Fig. 7C), suggesting GlcCer catabolism is reduced in LRRK2-PD patients. Given our findings that LRRK2 regulates BMP as a secondary response to its effects on GCase activity, we hypothesized that elevations in the ratio of total GlcCer to Cer might correlate with alterations in BMP in CSF from LRRK2-PD patients. We found a strong inverse correlation (r^2^ = 0.72, *p* < 0.001, Pearson correlation) between the levels of BMP(22:6/22:6) and the ratio of GlcCer to Cer levels in CSF from PD subjects that carry a *LRRK2* variant (Fig. 7D). Together, our results demonstrate that alterations in BMP correlate with reduced GlcCer catabolism in CSF from LRRK2-PD patients and suggest that LRRK2-dependent effects on GCase activity may drive BMP alterations in disease.

The identification of a CSF-based biomarker of LRRK2 activity and downstream pathway modulation is a key barrier in the development of LRRK2-targeted therapies. Such a biomarker could potentially be used for multiple valuable applications, including quantification of LRRK2 inhibition in the CNS to inform dose selection, measurement of a downstream lysosomal treatment response in CNS, or selection of patients that could be responsive to LRRK2-targeted therapies [70]. Based on our preclinical data and observations in CSF from LRRK2-PD patients, we explored the potential utility of BMP and GSLs as treatment-responsive biomarkers of LRRK2 inhibition in human subjects. We investigated the impact of the LRRK2 inhibitor DNL201 on these biomarkers in PD patients that carry a variant in LRRK2 in a phase 1b, double-blind, placebo-controlled study. Subjects were administered placebo (*n* = 3) or 50 mg of DNL201 (*n* = 4) three times daily, and CSF samples were collected at baseline and after 28 days of treatment [14]. We demonstrated that this level of LRRK2 inhibition led to a significant reduction in BMP levels in urine from subjects dosed with DNL201, suggesting improvement of peripheral lysosomal pathway defects in these subjects [14, 32]. Exploratory analyses of CSF samples revealed a trend toward reduced levels of GalCer and GlcCer in LRRK2-PD patients following DNL201 treatment, while BMP levels did not show a significant treatment response in CSF (Fig. 7E and Supplementary Fig. S7C). The lack of effect of LRRK2 inhibition on BMP levels in CSF is consistent with our data showing that BMP levels were unchanged in *LRRK2* KO mouse brain, suggesting that BMP may not be a sensitive biomarker of LRRK2 activity in CSF. These data suggest that GlcCer and GalCer levels could provide insight into the impact of LRRK2 inhibition on lysosomal function in the CNS. However, additional replication in larger cohorts of LRRK2-PD patients treated with LRRK2 kinase inhibitors is required to confirm GSLs as potential CSF biomarkers of LRRK2 activity in the clinic.

## Discussion

Variants in LRRK2 associated with increased kinase activity are proposed to contribute to PD pathogenesis, and growing evidence suggests LRRK2 activity is similarly elevated in patients with sporadic disease, suggesting this may represent a central node of dysfunction in PD [17, 71]. While kinase-activating LRRK2 variants have been shown to perturb lysosomal function [72, 73], many questions remain about how LRRK2 modulates the endolysosomal system and whether such regulation is relevant in PD. Our study reveals that LRRK2 regulates the lysosomal pathway by controlling the levels of BMP, a lysosomal lipid important for lipase activities, and GSLs, including GCase substrates that accumulate in PD. Using preclinical models, including human iPSC-derived microglia, and CSF from LRRK2-PD patients, we establish the functional relevance of LRRK2-mediated effects on lysosomal homeostasis by demonstrating that LRRK2 modulates BMP and GSL levels in the CNS. Finally, LRRK2 kinase inhibition fully normalized BMP and GSL levels in lysosomes, restored lysosomal proteolysis in cellular models, and showed trends toward reduction of GSL levels in CSF from human subjects, highlighting the potential of LRRK2 kinase inhibition as a therapeutic approach to correct lysosomal dysfunction in PD.

Our data suggest that LRRK2 exerts its effects on GSL levels by modulating GCase activity within lysosomes. We demonstrated that LRRK2 regulates GCase activity in a kinase-dependent fashion, as we observed that pathogenic LRRK2 variants led to a decrease in lysosomal GCase activity and corresponding accumulation of GCase substrate levels that were normalized following LRRK2 inhibition. The precise mechanisms by which LRRK2 kinase activity regulates the endolysosomal activity of GCase remain poorly understood. We showed that the LRRK2 substrates Rab10 and Rab12 modulate GCase activity, and *RAB12* deletion leads to GCase substrate accumulation (with a trend toward GlcCer elevation in *RAB10* KO cells). Rab12 was also recently identified as a key regulator of LRRK2 activity and lysosomal localization, suggesting that Rab12 can act both upstream and downstream of LRRK2 to mediate is effects on lysosomal function [28, 74]. Given that Rab GTPases function as master regulators of intermembrane trafficking, LRRK2 hyperactivity could in theory impact the intracellular trafficking of GCase itself or of a cofactor that plays a key role in promoting lysosomal GCase activity via phosphorylation of Rab10 and Rab12 [75–77]. Our data showed that the lysosomal levels of GCase were comparable between WT and LRRK2 R1441G cells, suggesting that the reduced GCase activity associated with LRRK2 hyperactivity and Rab10 or 12 deletion is not due to impaired delivery of GCase to the lysosome. We observed that lysosomes were hyper-acidified in cells expressing a pathogenic variant of LRRK2, and LRRK2 hyperactivity and increased Rab10 and/or Rab12 phosphorylation may lower endolysosomal pH into a range that is sub-optimal for GCase activity. Additional studies are warranted to define how LRRK2 activity regulates endolysosomal pH, what role Rab10 and 12 may play in this process, and whether this drives the observed defects in GCase activity. Further, the current study largely employed LRRK2 R1441G cells to assess the mechanisms by which LRRK2 hyperactivity impairs lysosomal function, and these results were compared to effects observed in LRRK2 G2019S KI iPSC-derived microglia, mice, and human subjects that carry the LRRK2 G2019S variant. Future work is needed to determine whether the molecular mechanisms uncovered here using LRRK2 R1441G KI cells are also relevant in the context of the LRRK2 G2019S variant.

We propose that LRRK2 regulates BMP levels as part of a compensatory response to perturbed lysosomal function and can employ multiple mechanisms to modulate BMP and ultimately restore GSL homeostasis depending on the cell type and tissue. In kidney, our data suggest that LRRK2 can reduce BMP and GSL accumulation by controlling their secretion. BMP and GSLs are enriched in ILVs within multivesicular bodies (MVBs) and can be secreted into the extracellular space when MVBs fuse with the plasma membrane in the form of exosomes [52]. We demonstrate that LRRK2 hyperactivity leads to a reduction in intracellular BMP and GSL levels in mouse kidney with a corresponding increase in BMP levels in urine from both mouse models and human subjects. Conversely, LRRK2 deletion or inhibition leads to the intracellular accumulation of BMP and GSLs along with their depletion from urine. The mechanism by which LRRK2 controls the secretion and/or content of extracellular vesicles remains unclear, but a tantalizing possibility is that this may occur through LRRK2’s known kinase activity on specific Rab GTPases. Indeed, several Rab GTPases, including the LRRK2 substrate Rab35, have been shown to directly impact the biogenesis or secretion of exosomes, and future studies are warranted to determine whether LRRK2-dependent Rab phosphorylation mediates the effects of LRRK2 activity on extracellular vesicle release [78, 79]. Emerging data suggest that exosome secretion may be employed as a homeostatic response to lysosomal damage to aid in the removal of lysosomal proteins and lipids that are not effectively degraded [51, 80, 81]. In other cell types, including microglia, we propose that LRRK2 hyperactivity can increase BMP levels within lysosomes as a direct consequence of its negative impact on GCase activity. We demonstrated that LRRK2 pathogenic variants led to reduced GCase activity and increased lysosomal BMP in A549 cells and iPSC-derived human microglia and showed that treatment with exogenous GCase was sufficient to reduce LRRK2-dependent BMP accumulation. There is precedent for alterations in BMP levels or composition resulting from deficient enzyme activity and primary storage across many LSDs, including in Gaucher disease [82–86]. BMP is thought to accumulate either as a secondary effect of broader impairments in lysosomal function and lipid catabolism or as an adaptive response to boost lipase activity and help degrade accumulated GSLs [83]. LRRK2 may normally function to sense and respond to defects in lysosomal proteolysis and lipid catabolism by triggering exosome secretion or the upregulation of lysosomal BMP levels in an effort to restore lysosomal homeostasis. Prolonged lysosomal dysfunction and chronic LRRK2 activation may impair the cell’s ability to respond to additional lysosomal damage and ultimately lead to GSL accumulation and secondary lipid storage.

Urinary BMP has been effectively used as a biomarker to assess LRRK2-dependent lysosomal changes in the periphery in ongoing clinical studies, but a robust CSF-based biomarker of LRRK2 activity has yet to be identified and represents a critical gap in assessing the impact of LRRK2 inhibitors in the CNS. While BMP levels were significantly accumulated in *LRRK2* KO mouse kidney and depleted in urine, BMP levels were not grossly impacted by LRRK2 throughout the mouse brain or in CSF from LRRK2-PD patients following dosing with the LRRK2 inhibitor DNL201. These data suggest that BMP may not be a useful biomarker of LRRK2 activity in the CNS. However, our findings identify GSLs, including GlcCer and GalCer, as potential CNS biomarkers of LRRK2-dependent lysosomal dysfunction. We observed broad alterations in several classes of GSLs, including GalCer, GlcCer, and sulfatides in CNS cells isolated from *LRRK2* KO and G2019S mice and detected elevated GlcCer levels in CSF from PD patients carrying a LRRK2 variant. Data from a phase 1b study assessing the impact of LRRK2 inhibition in a small cohort of LRRK2-PD patients also revealed a trend toward reduction in the levels of GlcCer and GalCer species in CSF following dosing with DNL201, highlighting the potential of GlcCer and GalCer as CNS biomarkers of LRRK2 inhibition. While the reductions in GlcCer and GalCer species in CSF observed following LRRK2 inhibition will require confirmation in a larger clinical study, our data provide support for the continued exploration of GSLs as PD-relevant biomarkers of LRRK2 activity in CSF.

Our results demonstrate that inhibition of LRRK2 kinase activity can normalize GCase substrate accumulation and downstream deficits in lysosomal function and highlight the therapeutic potential of LRRK2 inhibitors to correct lysosomal dysfunction observed in PD. Preclinical studies have shown that LRRK2 kinase inhibition can rescue lysosomal dysfunction not directly driven by kinase-activating mutations in LRRK2 [14, 63, 87, 88]. Variants in *GBA1* are observed in approximately 10% of PD patients, and increasing data, including that reported in the present work, suggest that GCase activity is impaired in PD patients beyond those that carry deleterious *GBA1* variants [8, 89–92]. Our work demonstrates that treatment with DNL151 can rescue lysosomal defects stemming from reduced GCase activity and provides additional preclinical support for the idea that LRRK2 inhibition may normalize lysosomal dysfunction more broadly in PD and could provide benefit to others beyond pathogenic LRRK2 variant carriers. Interestingly, human subjects that carry both the LRRK2 G2019S variant and an additional variant in *GBA1* do not have a worse clinical course of PD than those that carry a variant in either gene and, in fact, may have a beneficial effect with respect to cognitive decline [93]. DNL151 is currently being tested in late-stage clinical studies in both LRRK2-PD and sporadic PD patients, and the question of whether LRRK2 kinase inhibition can rescue PD-relevant defects in lysosomal homeostasis and modify disease progression in these patient populations will ultimately be resolved in the clinic.

## Supplementary Information

Below is the link to the electronic supplementary material.

Supplementary Material 1

## Data Availability

The preclinical data that support the findings of this study are available upon reasonable request. Urine BMP is available through PPMI (Project ID: 145). WGS data from PPMI is available through AMP-PD (https://www.amp-pd.org/register-for-amp-pd). De-identified individual clinical trial data that underlie the results reported in this manuscript will be made available to anyone who wishes to access the data for any purpose for up to 5 years after the publication of the article. To gain access, the data requester should email the corresponding author at henry@dnli.com Analysis code for the human genetics study of urine BMP levels and lipidomic analysis will be made available via Zenodo upon publication.

## Declarations

M.T.M., X.W., R.G., S.V.A., R.M., S.T.M., M.A., G.A., V.V.B, J.C., C.C., S.S.D., A.C.H., H.N.N., N.E.P., O.B.D., G.D.P., A.A.E., J.V., J.W.L., A.A., J.H.S., S.H.R., and A.G.H. were full time employees and shareholders of Denali Therapeutics during the course of this work. X.W. M.A., A.A.E. and J.D.V. are currently employees of Tenvie Therapeutics.

## Abbreviations

PD: Parkinson’s disease
BMP: Bis(monoacylglycerol)phosphate
GSL: Glycosphingolipids
GCase: b-glucocerebrosidase
CSF: Cerebrospinal fluid
LSDs: Lysosomal storage disorders
CD: Crohn’s disease
ILVs: Intraluminal vesicles
GlcCer: Glucosylceramide
GalCer: Galactosylceramide
WT: Wildtype
LPG: Lysophosphatidylglycerol
PG: Phosphatidylglycerol
IMS: Imaging mass spectrometry
WGS: Whole-genome sequencing
PPMI: Parkinson’s Progression Markers Initiative
pT73 Rab10: Phosphorylated T73 Rab10
EVs: Extracellular vesicles
FACS: Fluorescence-activated cell sorting
Lyso-IP: Lysosome immunoprecipitation
LysoFQ-GBA: Lysosomal GCase activity
iMicroglia: iPSC-derived microglia
GlcSph: Glucosylsphingosine
ASAH1: Acid ceramidase
CBE: Conduritol β-epoxide
LacCer: Lactosylceramide
Cer: Ceramide
MVBs: Multivesicular bodies
