# Supplementary material for: LRRK2 kinase activity regulates Parkinson’s disease-relevant lipids at the lysosome
Source: Mol Neurodegener. 2025 Aug 6;20:89. doi: 10.1186/s13024-025-00880-7 (PMC12330094; doi:10.1186/s13024-025-00880-7)
Supplement: Supplementary file 1 — Supplementary Material 1 [file 13024_2025_880_MOESM1_ESM.docx]

**Supplementary Information for: LRRK2 Kinase Activity Regulates Parkinson’s Disease-Relevant Lipids at the Lysosome**

**Authors:** Michael T. Maloney^1^*, Xiang Wang^1,4^*, Rajarshi Ghosh^1^, Shan V. Andrews^1^, Romeo Maciuca^1^, Shababa T. Masoud^1^, Maayan Agam^1,4^, Richard M. Caprioli^2^, Giuseppe Astarita^1^, Vitaliy V. Bondar^1^, John Chen^1^, Chi-Lu Chiu^1^, Sonnet S. Davis^1^, Audrey Cheuk-Nga Ho^1^, Hoang N. Nguyen^1^, Nicholas E. Propson^1^, Michelle L. Reyzer^2^, Oliver B. Davis^1^, Matthew C. Deen^3^, Sha Zhu^3^, Gilbert Di Paolo^1^, David J. Vocadlo^3^, Anthony A. Estrada^1,4^, Javier de Vicente^1,4^, Joseph W. Lewcock^1^, Annie Arguello^1^, Jung H. Suh^1^, Sarah Huntwork-Rodriguez^1^, Anastasia G. Henry^1#^

**Affiliations:**

^1^ Denali Therapeutics Inc., 161 Oyster Point Blvd., South San Francisco, CA 94080, USA.

^2^ Mass Spectrometry Research Center, Vanderbilt University, 9160 MRB III, 465 21 Avenue South, Nashville, TN 37240, USA.

^3^ Department of Chemistry, Simon Fraser University, Burnaby, BC V5A 1S6, Canada

^4^Current address: NICO Therapeutics, South San Francisco, CA 94080, USA.

*These authors contributed equally to this work

^#^To whom correspondence should be addressed: henry@dnli.com


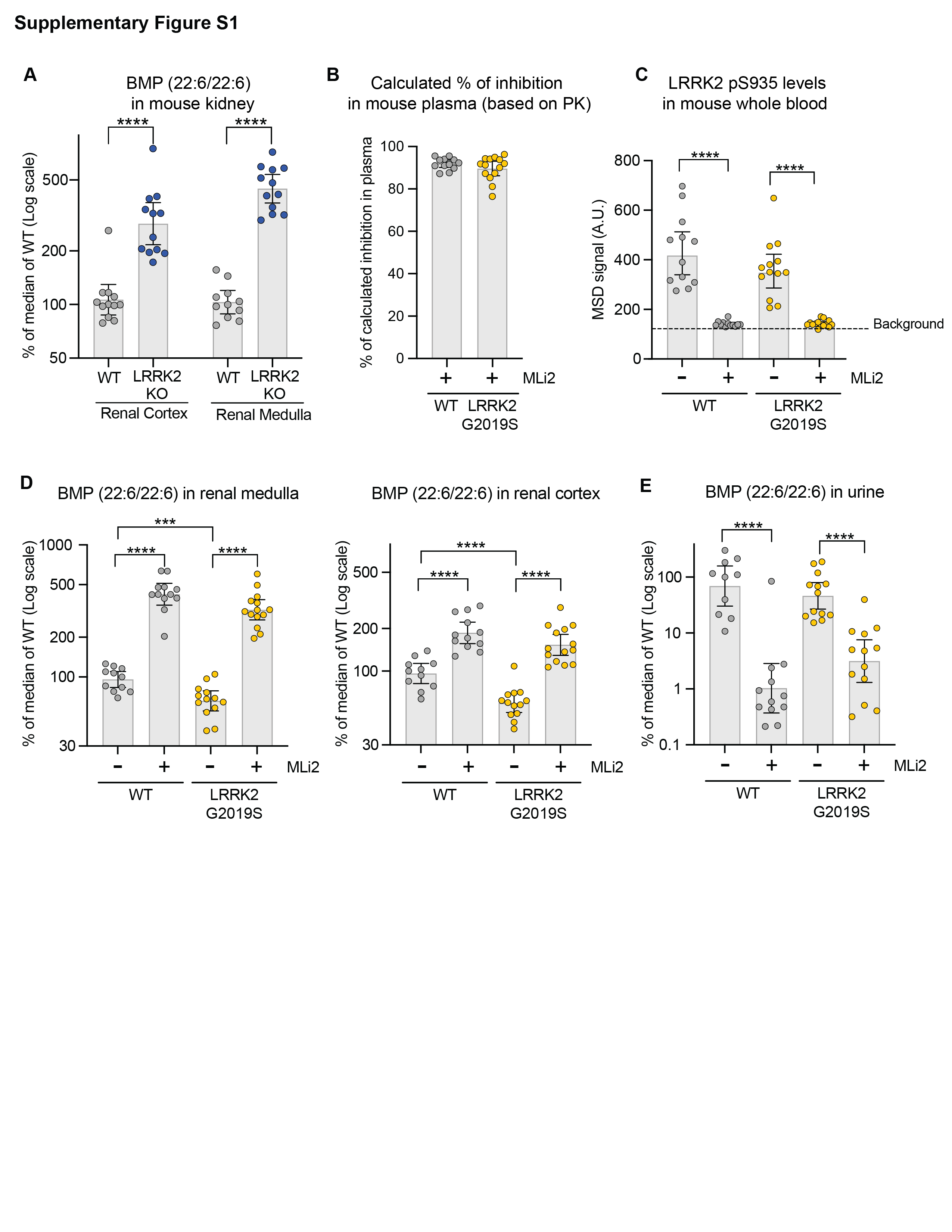


**Supplementary Figure S1: LRRK2 activity regulates BMP secretion in mouse kidney and urine**. A) The relative abundance of BMP(22:6/22:6) in the renal cortex and medulla from LRRK2 KO mice and WT littermates was measured by LC-MS/MS and normalized as a percent of median values of the WT group; n=11-12 animals for each group. Data are shown as geometric mean ratio (%) and 95% confidence intervals with statistical significance assessed based on Benjamini-Hochberg (BH)-adjusted p-values. B and C) Pharmacokinetics and pharmacodynamic analysis demonstrated >90% of LRRK2 kinase inhibition in the periphery in WT and LRRK2 G2019S KI mice dosed with MLi-2. B) Calculated % of inhibition based on the unbound concentration of the drug in plasma demonstrated >90% inhibition. C) The levels LRRK2 pS935 measured by MSD assay in whole blood showed significant reduction in WT and LRRK2 G2019S KI mice. Representative plots of BMP(22:6/22:6) in renal medulla and renal cortex (D) and in urine (E) from LRRK2 G2019S KI mice and WT littermates treated with or without MLi-2. Data are presented as % of the median values of WT-vehicle group and shown as geometric mean with 95% CI with p-values based on an ANCOVA model and statistical significance assessed at nominal levels. ***p ≤ 0.001, ****p ≤ 0.0001.

**
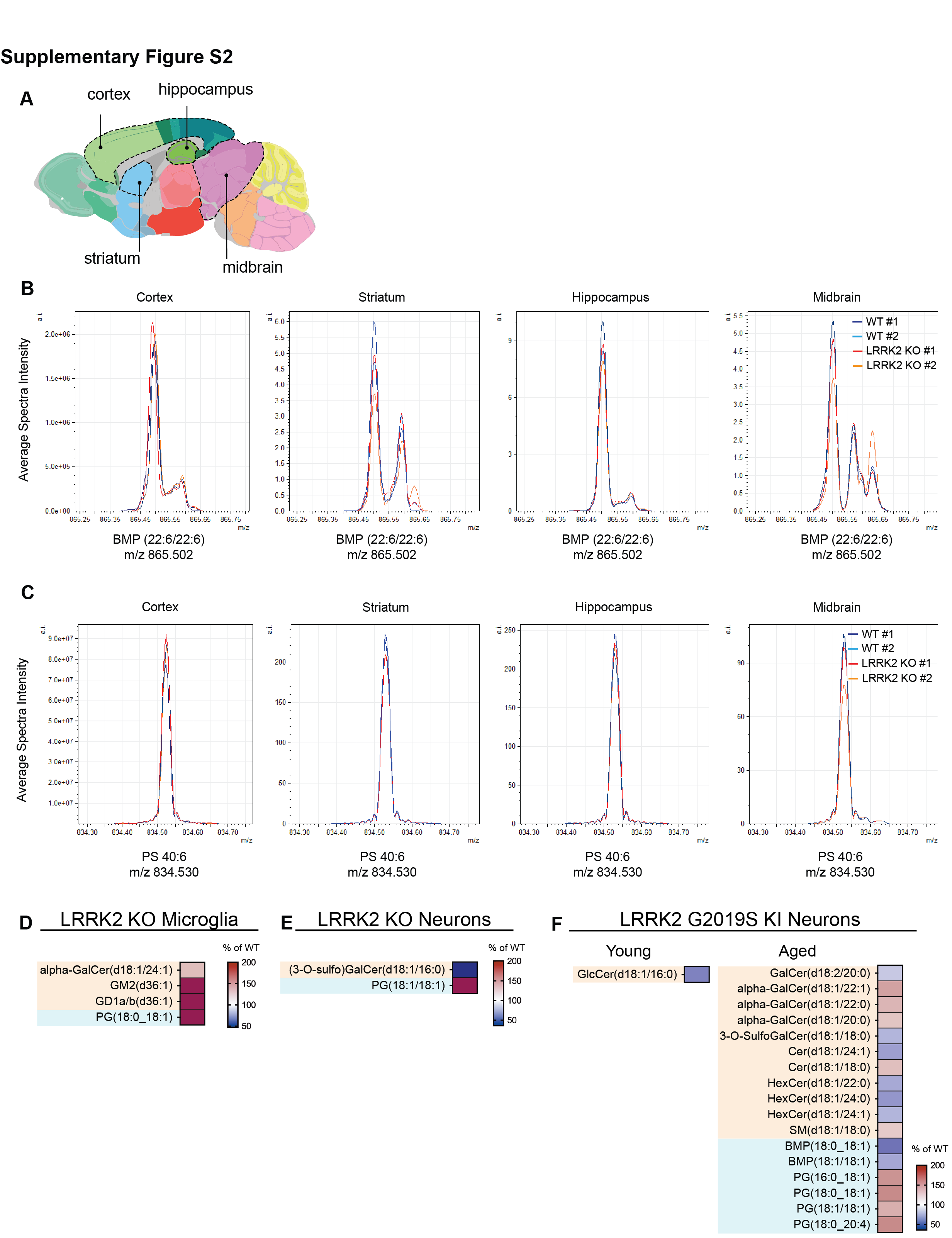
**

**Supplementary Figure S2: LRRK2 activity regulates glycosphingolipids in mouse brain and mildly impacts in BMP in cell-type specific manner.** A) Schematic indicating approximate location of sagittal brain regions of interest (ROIs) for comparison of average spectra intensity in Fig. S2B and S2C. Spectra were averaged across each brain ROI from the mass spectrometry imaging experiments. Exported spectra at a mass/charge ratio (m/z) of (B) 865.502, corresponding to BMP(22:6/22:6) and (C) 834.530, corresponding to PS(40:6) are shown for selected ROIs. Data presented includes n=2 mice per genotype with each color representing a biological replicate. Spectra show no significant differences in the intensities of BMP(22:6/22:6) or PS(40:6) in any of the selected brain regions. D-F) Heatmaps demonstrated the percent change in the levels of GSLs and BMP-related lipids in microglia from *LRRK2* KO mice (D), neurons from *LRRK2* KO mice (E), and neurons from LRRK2 G2019S KI mice at 5-6 months-old and 18-months old (F) compared to WT littermate controls. The analytes included had nominal p-values ≤ 0.10 for the genotype difference and were then grouped based on lipid class. The BMP-related lipids were shaded in cyan, and the GSL species were shaded in orange. In the color scale, white depicts levels detected in WT mice set to 100%, red shows an accumulation (capped at 200%) and blue shows a reduction.

**
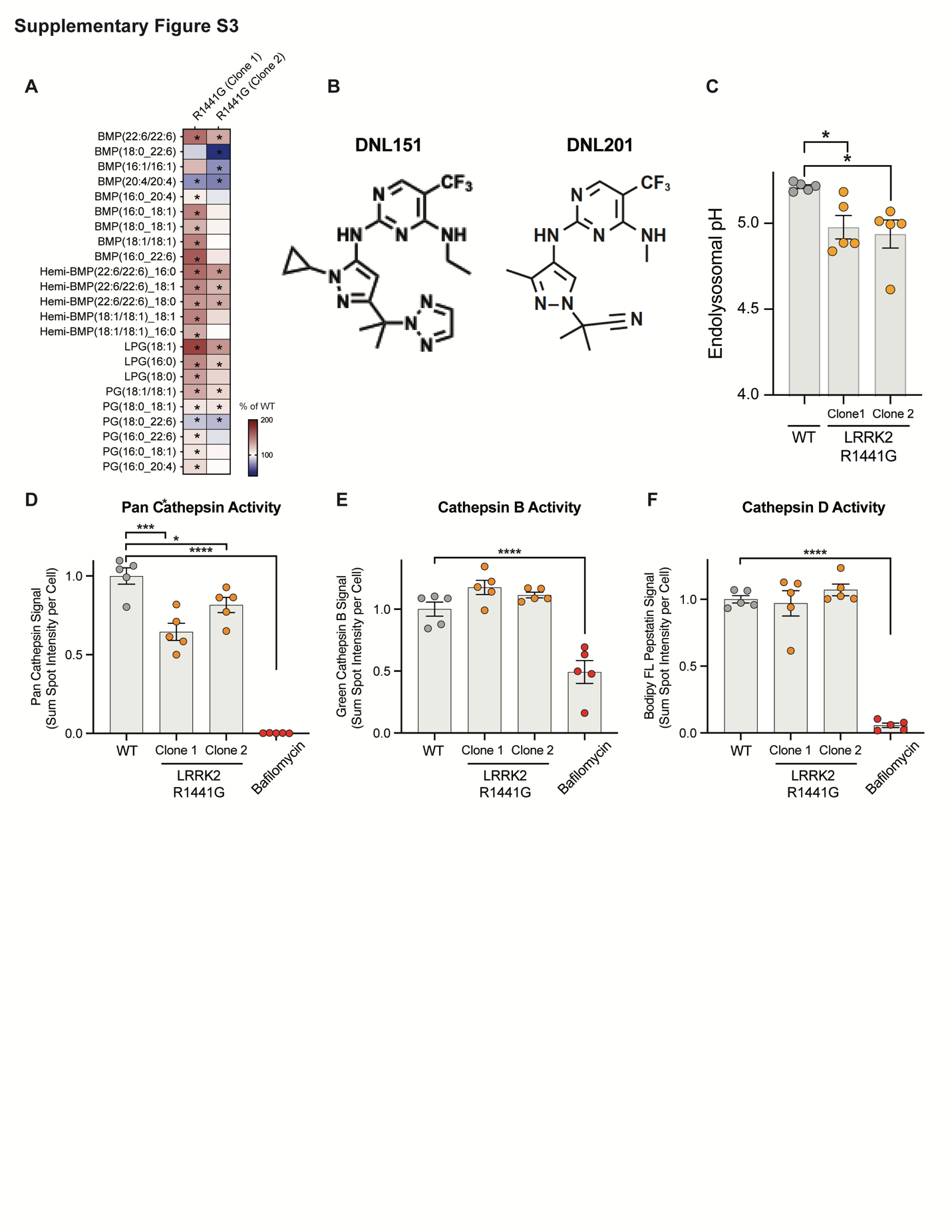
Supplementary Figure S3: Characterization of BMP-relevant lipids and endolysosomal function in LRRK2 R1441G KI A549 cells.** A) Heatmap showing alterations in BMP and BMP-related lipid species in whole cell extracts from two clones of LRRK2 R1441G KI A549 cells as compared to WT cells; n=14 independent experiments. B) Chemical structure of DNL151 and DNL201. C) Endolysosomal pH in WT and two clones of LRRK2 R1441G A549 cells was determined using the ratio of Oregon Green Dextran and TMR Dextran. Data are shown as mean ± SEM; n=5 independent experiments, and statistical significance was determined using one-way ANOVA and Dunnett method for multiple comparisons. D-F) Cathepsin endolysosomal activity was assessed using a pan-cathepsin activity-based probe (D), green fluorescent cathepsin B assay (E), and BODIPY FL Pepstatin A (F) in WT and two clones of LRRK2 R1441G A549 cells. Data are shown as mean ± SEM; n=5 independent experiments, and statistical significance was determined using one-way ANOVA and Dunnett method for multiple comparisons. * p ≤ 0.05, ***p ≤ 0.001, ****p ≤ 0.0001.


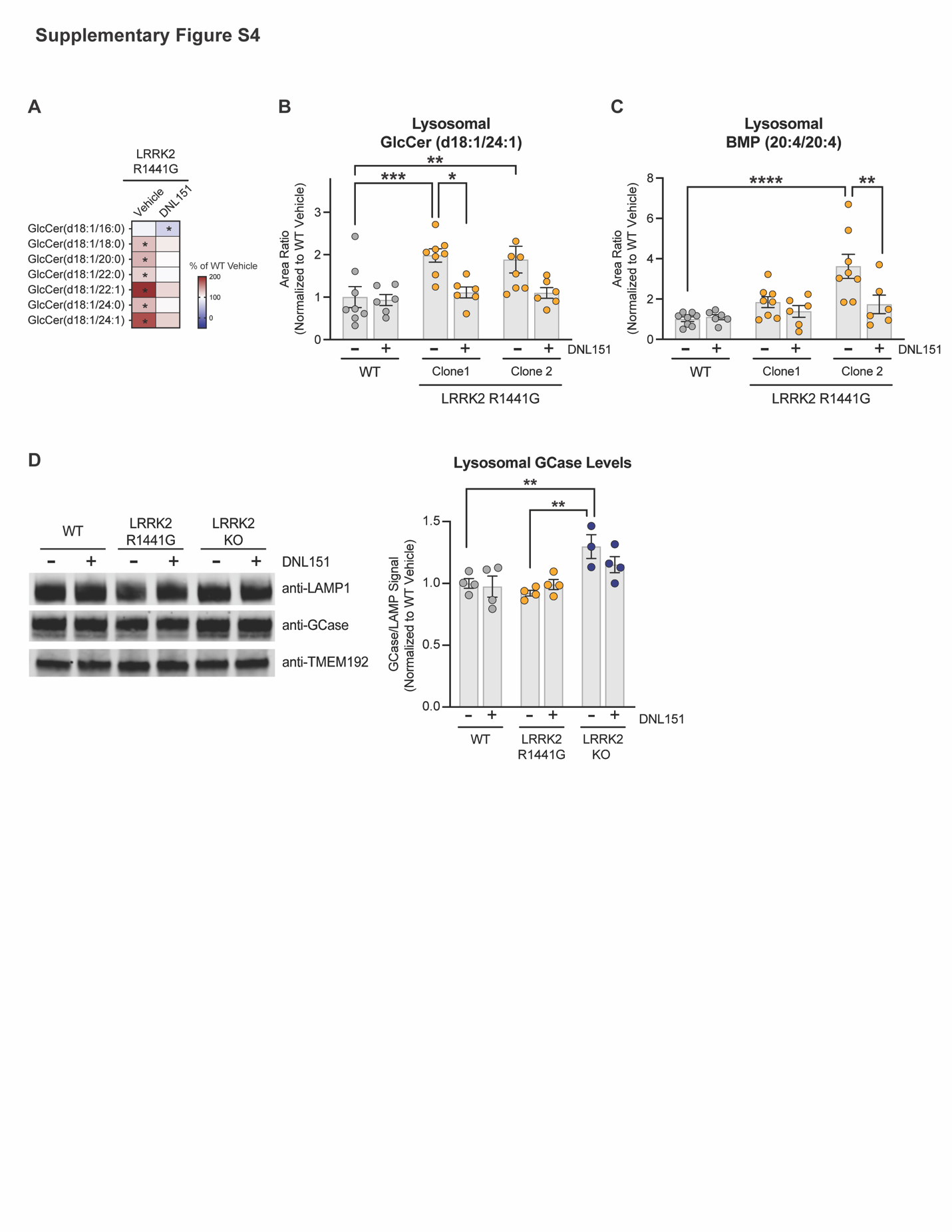


**Supplementary Figure S4: LRRK2 activity regulates lysosomal glycosphingolipid catabolism and BMP levels in A549 cells but does not impact lysosomal GCase levels.** A) Heatmap showing the levels of GlcCer species measured in lysosomes isolated from WT or one clonal line of LRRK2 R1441G KI A549 cells treated with vehicle or DNL151. The heatmap was generated as % change by normalizing the average of different groups to the average of the WT group. White in the color scale depicts the WT-vehicle amounts, as 100%, red shows an accumulation (capped at 200%), and blue shows a reduction. The analytes with nominal p-values ≤ 0.10 compared to WT cells treated with vehicle are denoted with an asterisk (*). B and C) WT and two clones of LRRK2 R1441G KI A549 cells were treated with either vehicle or DNL151 (2μM) for 72 hours, lysosomes were immunoprecipitated, and the levels of GlcCer(d18:1/24:1) or BMP(20:4/20:4) were measured using LC-MS/MS-based analysis. Data are shown as mean ± SEM; n = 6-8 independent experiments, and statistical significance was determined using one-way ANOVA following log transformation. D) The levels of GCase in lysosomes isolated from WT, one clonal line of LRRK2 R1441G, and one clonal line of LRRK2 KO cells were assessed by western blot analysis using antibodies against GCase, HA (to detect TMEM192-3x-HA), and LAMP1. The GCase signals were normalized to the LAMP signal, then were normalized to the median within each experimental replicate, and expressed as a fold change compared to lysosomes isolated from WT A549 cells treated with vehicle. Data, error bars and statistical analysis are rendered as described in the previous panels; n=3-4 independent experiments; *p ≤ 0.05. ** p ≤ 0.01, ***p ≤ 0.001, ****p ≤ 0.0001.

**
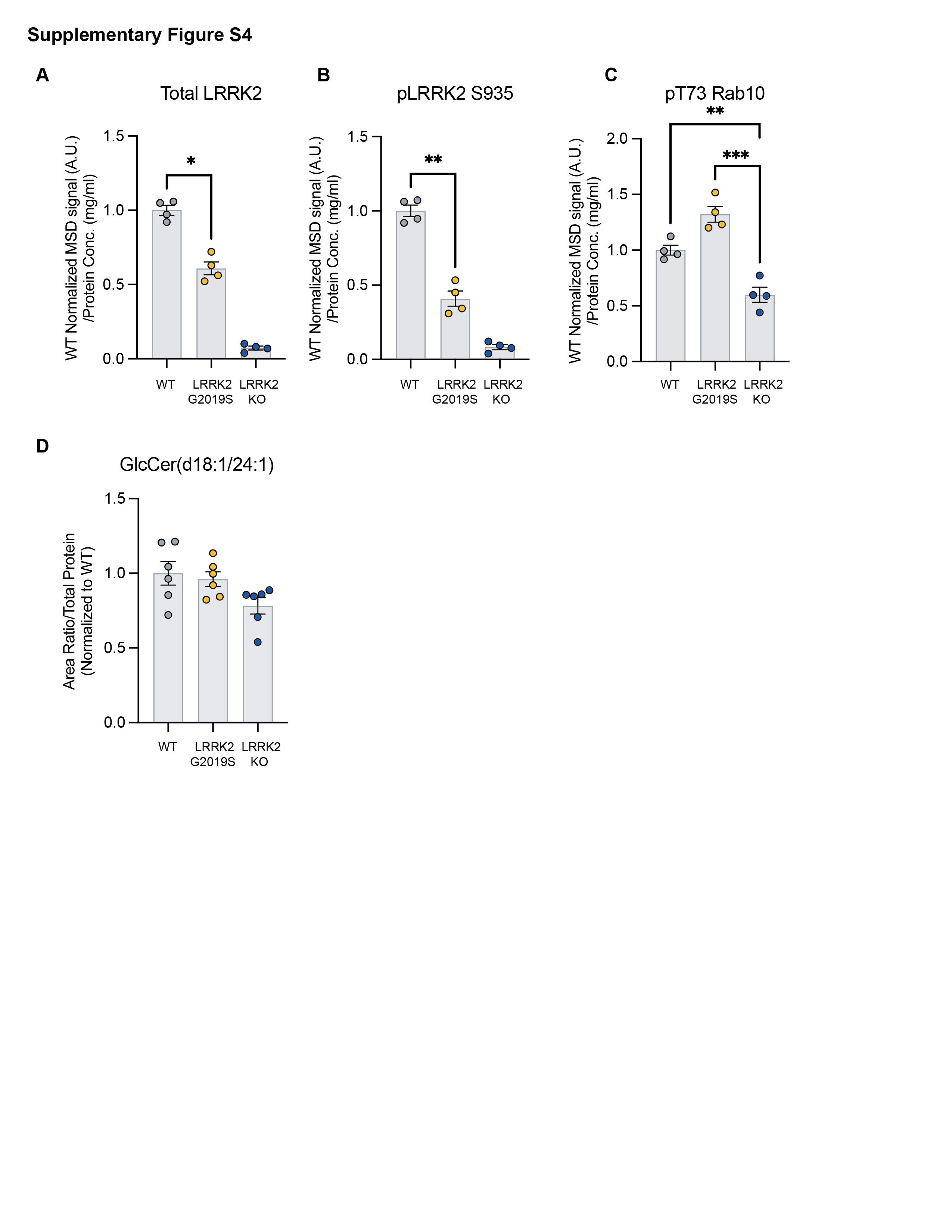
Supplementary Figure S5: Analysis of LRRK2 levels, Rab10 phosphorylation, and GlcCer levels in LRRK2 G2019S and KO iMicroglia.** A) Total LRRK2, (B) pS935 LRRK2 and (C) pT73 Rab10 protein levels were measured in WT, LRRK2 G2019S and *LRRK2* KO iMicroglia cell lysates using MSD-based assays; n=4 independent experiments; one-way ANOVA, Tukey’s method for multiple comparisons. D) LC-MS/MS-based analysis of GlcCer species shows no significant changes across the different *LRRK2* genotypes in iMicroglia; n=6 independent experiments. Data are shown as geometric mean ± SEM; * p ≤ 0.05, **p ≤ 0.01, ***p ≤ 0.001.

**
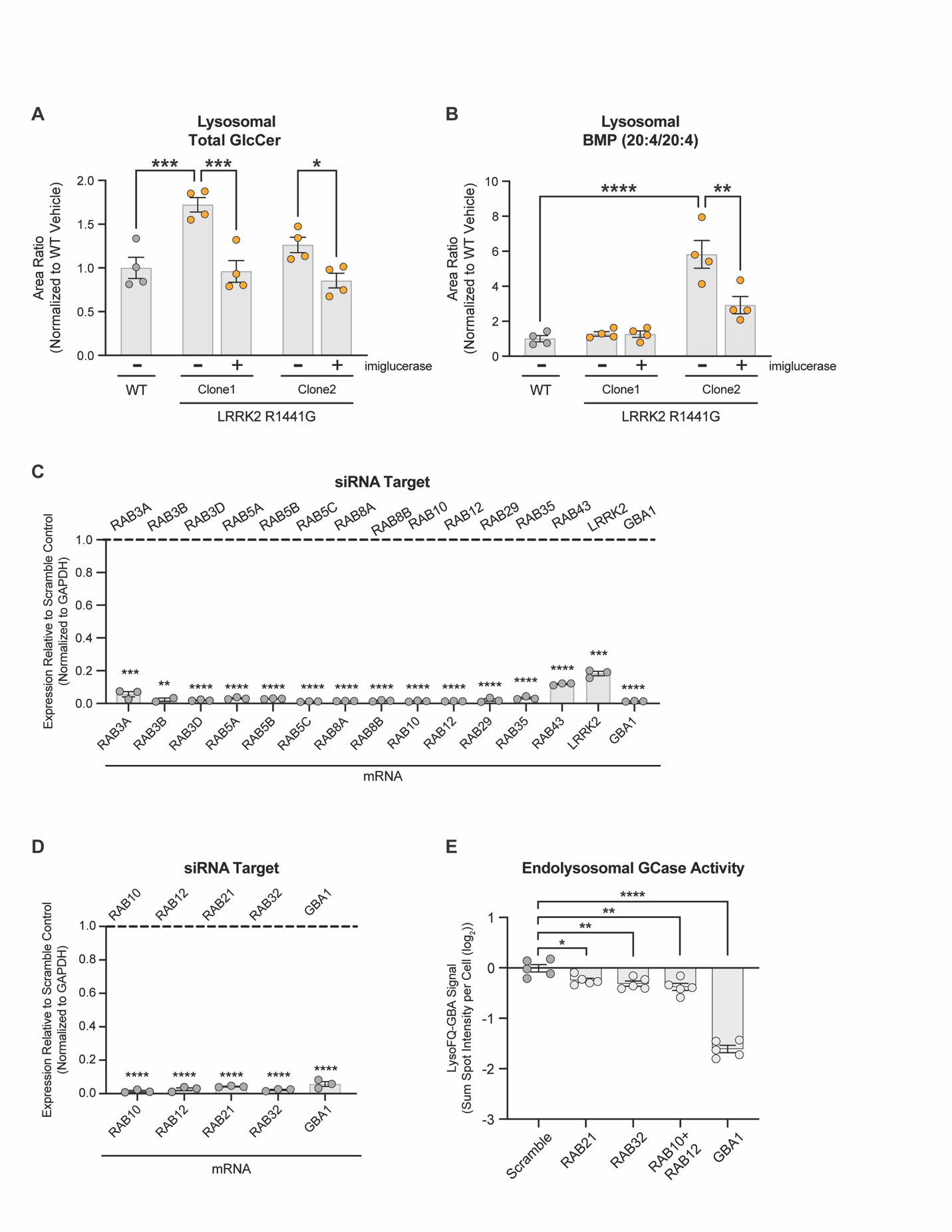
**

**Supplementary Figure S6: Characterization of effects of exogenous GCase on lysosomal levels of GlcCer species and BMP in two LRRK2 R1441G KI clones and confirmation of siRNA-mediated KD of LRRK2-Rab substrates.** A and B) WT and two clones of LRRK2 R1441G KI A549 cells were treated with vehicle or imiglucerase (2μM) for 72 hours, and the levels of total GlcCer and BMP(20:4/20:4) were measured in lysosomes from these cells using LC-MS/MS-based analysis. Data are shown as mean ± SEM; n=4 independent experiments, and statistical significance was determined using one-way ANOVA following log transformation. **C)** A549 cells were transfected with siRNA targeting LRRK2, GBA1 and LRRK2-Rab substrates, and knock-down was confirmed by qPCR-based analysis. The expression of each gene assessed was normalized to GAPDH expression, and then normalized to the expression observed with a scramble siRNA. Data are shown as the mean ± SEM, n=3 independent experiments, and statistical significance was determined by paired t-test. D and E) Rab expression was transiently knocked down in WT A549 cells using transfection of pooled targeted siRNAs, and GCase activity was evaluated using the live cell LysoFQ-GBA probe. The expression of each RAB gene or GBA1 was measured and normalized as described in (C). Data are shown as the mean ± SEM, n=5 independent experiments, and statistical significance was determined by one-way ANOVA with Tukey method for multiple comparison following log transformation; * p ≤ 0.05, **p ≤ 0.01, ***p ≤ 0.001, ****p ≤ 0.0001.


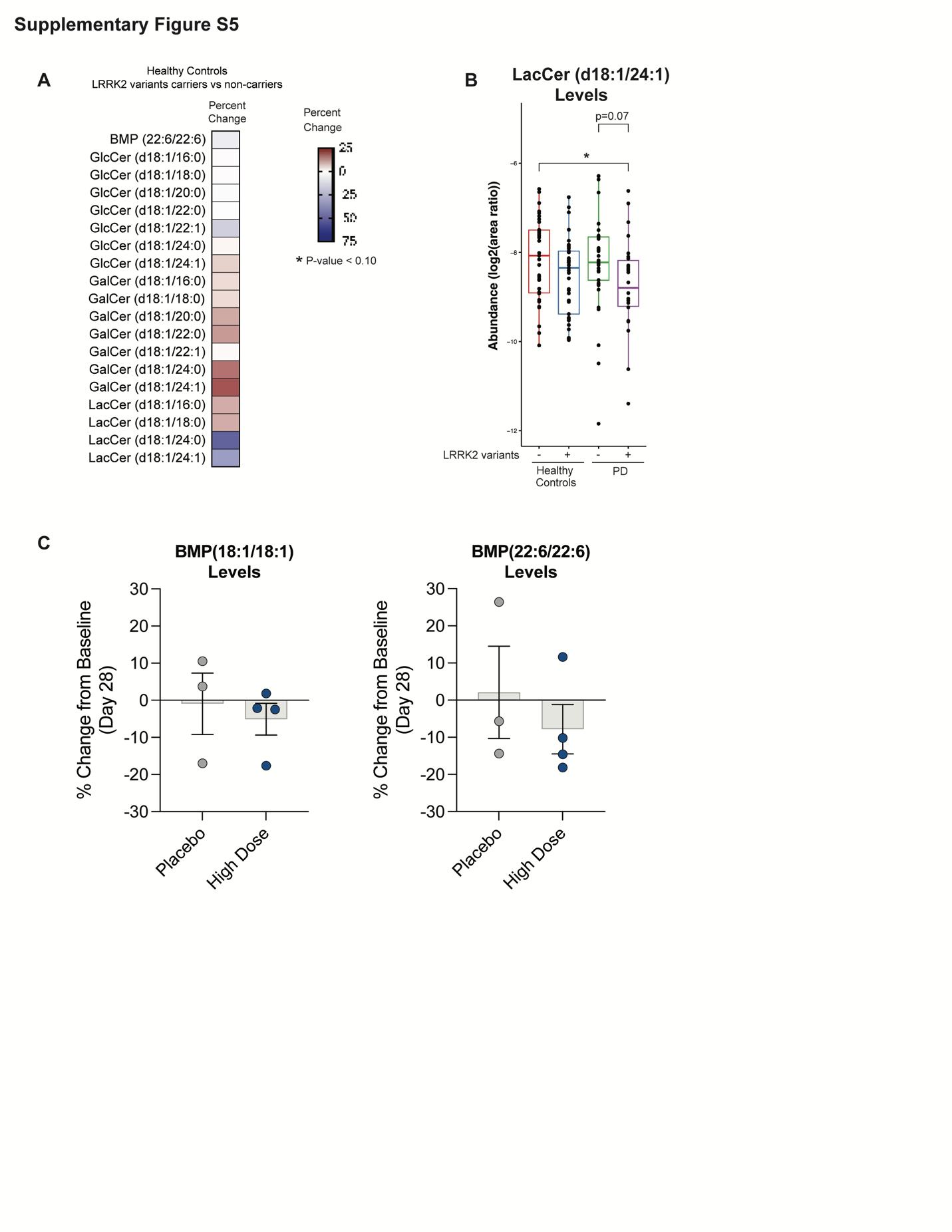


**Supplementary Figure S7: Analysis of BMP and GSL levels in human CSF from healthy subjects and PD patients with or without LRRK2 variants.** A) Heatmap showing % change in lipid abundance of BMP and GSL detected in human CSF from healthy subjects carrying LRRK2 variants compared to non-carriers. % changes and significance of effects were analyzed using robust linear model with sex and age as covariates. None of the analytes in the heatmap showed significant difference between the two groups, using unadjusted p ≤ 0.10 as cutoffs. B) Relative abundance of LacCer(d18:1/24:1) levels in CSF. Significance of change was analyzed by linear model with pairwise comparisons by Tukey’s honest significant difference test with significance set at unadjusted p value of 0.05. Main box and error bars depict interquartile ranges of top 75^th^ or bottom 25^th^ percentile and largest and smallest value with 1.5 times the interquartile ranges above and below 75^th^ or 25^th^ percentiles. Median 50^th^ percentile is shown as midline within each boxplot. C) The levels of BMP(18:1/18:1) and BMP(22:6/22:6) were measured in CSF from PD subjects that carry a *LRRK2* variant at baseline and following 28 days of dosing with placebo (n=3) or DNL201 (n=4). The percent change from baseline was analyzed by calculating the lipid abundance change from the day 28-post-dose to pre-dose-baseline and then normalizing the change to lipid abundance at pre-dose baseline. Data are shown as mean ± SEM; *p ≤ 0.05.

Note: p-values were rounded to 4 decimal points

**Supplementary Table S1: Targeted lipidomic analysis of BMP related lipids and glycosphingolipids in kidney from LRRK2 KO and G2019S KI mouse.** Lipids from a targeted panel were measured in by LC-MS/MS from renal cortex and renal medulla. The table included BMP related lipids and glycosphingolipids with BH-adjusted ANCOVA p-values ≤ 0.05 for the difference between KO Vs WT for table A, or nominal ANCOVA p-values ≤ 0.10 for the difference between G2019S KI Vs WT in table B.

**
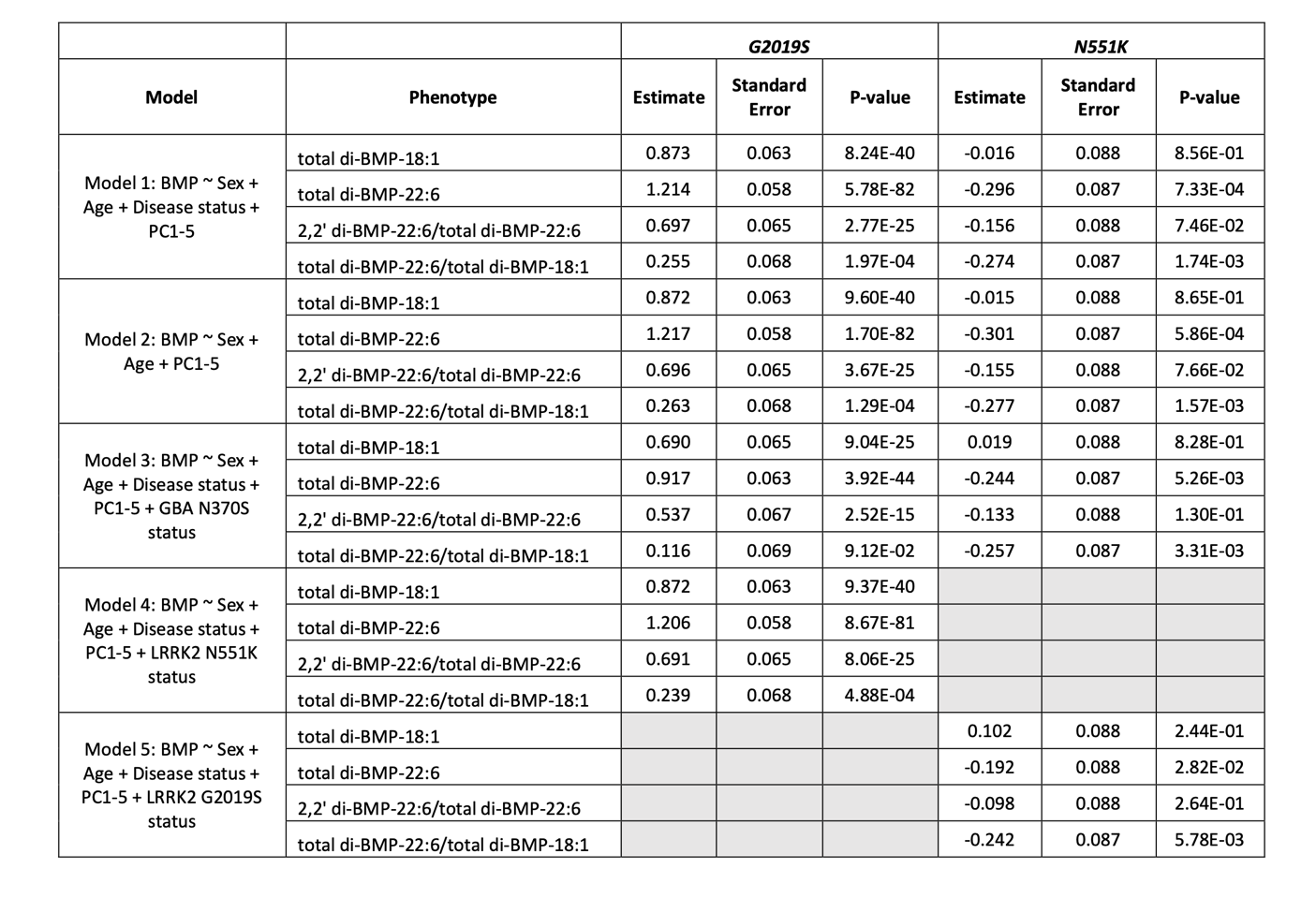
Supplementary Table S2: Linear regression statistics for association testing of G2019S and N551K status on urine BMP levels in PPMI.** Urine BMP measurements were normalized to creatinine, log transformed, and fit in a linear model against the covariates listed under each “Model”. Residuals from each model where then inverse normal transformed and tested for association against G2019S status and N551K status. Estimate = standard deviation change in adjusted and transformed BMP levels for dosage of each G2019S/N551K allele.

Note: p-values were rounded to 4 decimal points

**Supplementary Table S3: Targeted lipidomic analysis in CNS cells from LRRK2 KO and G2019S KI mice.** Lipids from a targeted panel were measured in by LC-MS/MS from FACS isolated astrocytes, microglia and neurons from LRRK2 mice. The table included BMP related lipids and glycosphingolipids with nominal p-values ≤ 0.10 for the genotype difference.

|  | LRRK2-  HC | LRRK2-  PD | LRRK2+  HC | LRRK2+  PD | Overall |
| --- | --- | --- | --- | --- | --- |
|  | (N=35) | (N=37) | (N=37) | (N=26) | (N=135) |
| Age |  |  |  |  |  |
| Mean (SD) | 53.0 (14.7) | 58.3 (11.5) | 50.0 (14.4) | 62.8 (11.5) | 55.5 (13.9) |
| Median  (Q1, Q3) | 54.0  (42.0, 64.0) | 59.0  (50.0, 67.0) | 50.0  (40.0, 61.0) | 65.0  (55.3, 70.3) | 57.0  (44.0, 66.0) |
| Min, Max | 24.0, 83.0 | 26.0, 77.0 | 27.0, 80.0 | 39.0, 80.0 | 24.0, 83.0 |
| Sex |  |  |  |  |  |
| Female | 19 (54.3%) | 13 (35.1%) | 17 (45.9%) | 13 (50.0%) | 62 (45.9%) |
| Male | 16 (45.7%) | 24 (64.9%) | 20 (54.1%) | 13 (50.0%) | 73 (54.1%) |

**Supplementary Table S4: Demographics of LRRK2 Cohort Consortium Participants with CSF Analyzed in this Study.** The demographics of the metabolomic and lipidomic CSF profiling study from the LRRK2 Cohort Consortium are summarized. HC: Healthy Control; PD: Parkinson’s disease; LRRK2-: Participants not carrying a LRRK2 pathogenic point mutation; LRRK2+: Participants carrying a LRRK2 pathogenic point mutation.

**Supplementary Methods**

*siRNA-Mediated Knockdowns of Rab GTPases*

A549 cells were transfected with Dharmacon SMARTpool siRNA targeting 4 Rab GTPases genes, GBA1 and non-targeting scramble control (Horizon Discovery, Cambridge, United Kingdom), using DharmaFECT 1 (Horizon T-2001-01). Cells were collected 3 days after transfection for mRNA analysis.

| Targets | Catalog Number |
| --- | --- |
| ON-TARGETplus Non-targeting Control | D-001810-10 |
| RAB10 | L-010823-00 |
| RAB12 | L-023375-02 |
| RAB21 | L-009450-00 |
| RAB32 | L-009920-00 |
| GBA1 | L-006366-00 |

*RT-PCR-Based Analysis of Rab Expression*

Total RNA was extracted from cells using RNeasy Plus Micro Kit (QIAGEN, Hilden, Germany, #74034). cDNA was synthesized from 1-2µg of RNA using Superscript IV VILO master mix (Thermo Fisher #11756050). The cDNA was diluted 3-fold and 1µL of diluted cDNA was used as template. To measure the relative expression levels of mRNAs by RT-qPCR, Taqman Fast Advanced Master Mix (Thermo Fisher #4444557) was used, together with gene specific primers using TaqMan Assays (Thermo Fisher). GAPDH was used as a housekeeping gene. The PCR reaction was run using QuantStudio^TM^ 6 Flex Real-Time PCR System, 384-well (Thermo Fisher). Gene expression was analyzed using 2^^(delta-delta Ct)^ method with GAPDH as an internal control.

| Taqman assay ID | Gene Name | Dye |
| --- | --- | --- |
| Hs00794658_m1 | Rab10 | FAM-MGB |
| Hs01391604_m1 | Rab12 | FAM-MGB |
| Hs00209226_m1 | Rab21 | FAM-MGB |
| Hs00199149_m1 | Rab32 | FAM-MGB |
| Hs00986836_g1 | GBA1 | FAM-MGB |
| Hs99999905_m1 | GAPDH | VIC |

*Live-Cell Cathepsin Activity Assays*

Briefly, cells were seeded into 96-well PDL-coated plates. As a negative control, bafilomycin A (CST 54645) was added to designated wells at a concentration of 1µM for 2 hours prior to addition of live-cell activity probes. Cells were treated with the following live-cell cathepsin activity-based probes for two hours under standard incubation conditions (humidified, 37^o^C, 5% CO2): Green Fluorescent Cathepsin B Assay (Antibodies Inc., 9512), BODIPY FL Pepstatin A (Fisher P12271), and IVISense Pan Cathepsin 680 Fluorescent Probe (Revvity). After two hours, cells were rinsed 3x with 37^o^C PBS, returned to standard culture medium, and counterstained with NucBlue (Invitrogen R37605) for ten minutes for labeling nuclei prior to imaging.

Imaging was performed on a Perkin Elmer Opera Phenix High Content Imaging System with acquisition of signal from the rhodamine cathepsin B probe R110-(RR)_2_ (ex:500nm/em:525nm), BODIPY Pepstatin A probe (ex: 502nm/em;511nm), pan cathepsin probe (ex:680nm/em:700nm), and NucBlue (ex:375nm/em 435-480nm) using a 40X water immersion objective. Analysis of cathepsin activity per cell was performed using Harmony software. Harmony Spot Analysis was used to identify probe-positive spots within the cell, to quantify the “corrected spot intensity”, and the sum of corrected spot intensities per field of view was normalized to total number of nuclei in the field (to account for differences in cell number) and as reported as “Sum Corrected Spot Intensity”.
